# Supplementary material for: Theoretical studies on anhydride dynamic covalent bond exchange mechanisms
Source: Commun Chem. 2025 Nov 17;8:351. doi: 10.1038/s42004-025-01736-3 (PMC12624005; doi:10.1038/s42004-025-01736-3)
Supplement: Supplementary file 2 — Supplementary Information [file 42004_2025_1736_MOESM2_ESM.pdf]

Supplementary Information

Theoretical studies on anhydride dynamic covalent bond exchange mechanisms

Xinglong Zhang,[1,2,]* Qiubo Chen,[1] Nannan Li,[1] Michael B. Sullivan,[1] Jianwei Zheng[1,]*

[1]Institute of High Performance Computing, Agency for Science, Technology and Research (A*STAR), 1 Fusionopolis Way, #16-16, Connexis, Singapore 138632, Singapore

[2]Department of Chemistry, The Chinese University of Hong Kong, Shatin, New Territories, Hong Kong, China

Email: xinglong.zhang@cuhk.edu.hk; zhengjw@a-star.edu.sg

Table of Contents

I. Computational Methods **2**

I.1 Benchmarking of prototypical system **2**

II. Mechanism of dynamic covalent bond exchange between methacrylic anhydride (MAA) and 4-pentenoic anhydride (PNA) **3**

II.1 Preliminary DFT benchmarking **3**

II.2 Concerted mechanism **7**

II.3 Relaxed potential energy surface (PES) scan **8**

II.4 Alternative mechanisms **9**

II.5 Conformational sampling **10**

II.6 Bond dissociation enthalpies (BDEs) **10**

II.7 Correlation with experimental data **11**

II.8 Mechanism under acid catalysis **14**

II.9 Effect of Temperature on Gibbs free energy correction **16**

II.10 Effect of inclusion of D3 correction in M06-2X in resulting Gibbs free energy profile **18**

II.11 Effect of Boltzmann weighting in resulting Gibbs free energy profile **18**

II.12 Frontier molecular orbitals, natural bond orbital (NBO) charges, non-covalent interaction (NCI) plots and distortion-interaction/activation-strain analyses **19**

III. DFT-optimised structures and absolute energies **26**

IV. Supplementary References: **37**

I. Computational Methods

I.1 Benchmarking of prototypical system

For the study of the mechanism of the prototypical system of anhydride exchange between methacrylic anhydride (MAA) and 4-pentenoic anhydride (PNA) molecules, the global hybrid DFT functional M06-2X[1] was employed. To test the effect of basis sets, the geometry optimization were performed in the gas phase separately with the Karlsruhe-family double-ζ valence def2-SVP[2,3] basis set for all atoms or with the Pople-type split-valence, singly diffuse basis set 6-31+G(d).[4] For the M06-2X/def-SVP gas phase optimised structures, single point (SP) corrections were performed using M06-2X functional and def2-TZVP[2,3] basis set for all atoms. For the M06-2X/6-31+G(d) gas phase optimised structures, SP corrections were performed using M06-2X functional and 6-311++G(d,p)[4] basis set for all atoms. For each of these SP calculations, the implicit SMD continuum solvation model[5] for chloroform ($CHCl_3$) solvent was used to account for the effect of solvent on the potential energy surface (PES). For a third method, geometry optimizations were carried out directly in implicit SMD solvent chloroform at M06-2X/def-SVP level of theory, followed by the SP correction in implicit SMD solvent chloroform at M06-2X/def-TZVP level of theory. In summary, three different levels of theories were tested: SMD($CHCl_3$)-M06-2X/def2-TZVP//M06-2X/def-SVP, SMD($CHCl_3$)-M06-2X/6-311++G(d,p)//M06-2X/6-31+G(d), SMD($CHCl_3$)-M06-2X/def2-TZVP//SMD($CHCl_3$)-M06-2X/def-SVP. These calculations were performed with *Gaussian 16* rev. B.01 software.[6]

Additionally, for the SP calculation on the gas phase optimised geometry at M06-2X/def-SVP level of theory, we performed the domain-based local pair natural orbital – coupled cluster with perturbative triple excitations (DLPNO-CCSD(T)) calculations[7,8] using ORCA version 5.0.4[9–11]. T_0 approximation which neglects the couplings between different triples by the off-diagonal Fock matrix elements, instead of the recently published iterative T_1 algorithm[12], was employed. The NormalPNO settings with $T_{cutPairs} = 10^{-4}$, $T_{CutDO} = 10^{-2}$, $T_{CutPNO} = 3.33\times10^{-7}$ and $T_{CutMKN} = 10^{-3}$ was used throughout. The TightSCF convergence with KDIIS algorithm[13] for SCF iterations were used. The complete basis set (CBS) extrapolation scheme of Helgaker et al.[14–16] using correlation-consistent double-/triple-ζ cc-pV(DT)Z basis set.[17–19] The auxiliary basis sets required for the integral evaluations in the DLPNO-CCSD(T) correlation energy calculations were generated automatically using the "AutoAux" command from the automated auxiliary basis set construction module[20] of ORCA. DEFGRID3 grid for integration was employed throughout.

For the extrapolation to complete basis set, the obtained values from cc-pVDZ basis set and from cc-pVTZ basis set are extrapolated according to the following formulae:

$$E_{\mathrm{SCF}}^{(X)} = E_{\mathrm{SCF}}^{(\infty)} + A\exp(-\alpha\sqrt{X}) \qquad \text{Eq (1)}$$

$$E_{corr}^{(\infty)} = \frac{X^{\beta}E_{\mathrm{corr}}^{(X)} - Y^{\beta}E_{\mathrm{corr}}^{(Y)}}{X^{\beta} - Y^{\beta}} \qquad \text{Eq (2)}$$

for the extrapolation of HF energy (Eq (1)) and of correlation energy (Eq (2)) to the basis set limit, respectively. $E^{(X)}_{\mathrm{SCF}/corr}$ is the SCF/correlation energy calculated with basis set of cardinal number X, and $E^{(\infty)}_{\mathrm{SCF}/corr}$ is the basis set limit SCF/correlation energy and A, α, and β are constants. For correlation energy, X and Y are the cardinal numbers of the basis sets used for extrapolation (X=2, Y=3 herein). For Extrapolate(2/3, cc), α=4.42 (SCF extrapolation), and β=2.46 (correlation extrapolation).

All structural and molecular orbital visualisations were performed using the open-source PyMOL[21] software.

II. Mechanism of dynamic covalent bond exchange between methacrylic anhydride (MAA) and 4-pentenoic anhydride (PNA)

Supplementary Scheme 1 shows the model reaction for the anhydride covalent bond exchange between methacrylic anhydride (MAA) and 4-pentenoic anhydride (PNA) used in our computational studies. This reaction has experimentally measured kinetic values using ^{13}C NMR spectroscopy in deuterated chloroform solvent, $CDCl_3$, for the anhydride dynamic covalent bond exchange.[22]

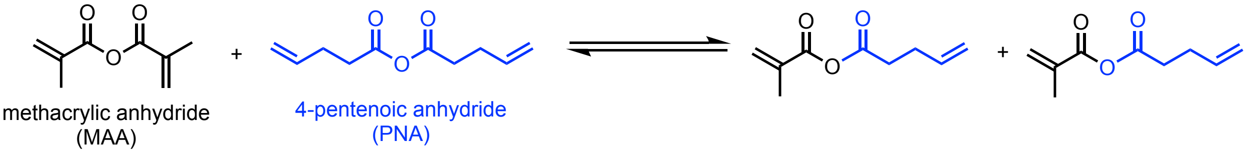

Supplementary Scheme 1. Model reaction for anhydride covalent bond exchange between methacrylic anhydride and 4-pentenoic anhydride.

II.1 Preliminary DFT benchmarking

Preliminary mechanistic studies located the 4-membered transition states (TSs) (Scheme 2ii, pathway b) and these are then used for benchmarking the different DFT bases on their effect on the PES. In studying this pathway, we kept all relative conformations and orientations of side groups consistent throughout. Note that although we later found a more favourable pathway via 6-membered TSs (Scheme 2ii, pathway a), for the purpose of initial benchmarking of DFT levels of theory, it is sufficient to consider any reaction pathway.

Four levels of theory were chosen for comparison: 1) SMD($CHCl_3$)-M06-2X/def2-TZVP//M06-2X/def2-SVP; 2) SMD($CHCl_3$)-M06-2X/def2-TZVP//SMD($CHCl_3$)-M06-2X/def2-SVP; 3) SMD($CHCl_3$)-M06-2X/6-311++G(d,p)//M06-2X/6-31+G(d) and 4) SMD($CHCl_3$)-DLPNO-CCSD(T)/CBS(complete basis set, cc-pVDZ, cc-pVTZ)//M06-2X/def2-SVP. We adopt the conventional DFT notation in which the separator "//" is used to separate the level of theory for geometry optimization that comes after it, and the level of theory for high-level single-point energy correction (usually together with implicit solvation model) that comes before it. A single "/" separator separates DFT functional that comes before it and basis set that comes after it. In comparing levels 1 and 2, we aim to discern if there is any effect using the geometry optimised in

the gas phase versus optimised in the solvent phase. In comparing levels 1 and 3, we aim to see if there is any effect of using different basis sets, namely the more modern Karlsruhe basis sets (def2-family) [2,3] vs. Pople basis sets[4]. Finally, comparing levels 1 and 4 allows us to see the difference of employing DFT-based method versus wavefunction-based method, domain-based local pair natural orbital–coupled cluster with perturbative triple excitations (DLPNO-CCSD(T)) calculations[7,8] for single point energy correction.

The pathway for the 4-membered, stepwise mechanism (*vide infra*) was initially used for benchmarking the potential energy surface (PES), Supplementary Figure 1. Within each level of theory, the Gibbs free energy for each species is taken relative to the sum of the Gibbs free energies of the reactants (MAA and PNA) (Supplementary Figure 1). We can see that the Gibbs free energies are quite similar (within 2–4 kcal mol^{-1}) whether we use the optimised geometries in the gas phase (level of theory 1) or the solvent phase (level of theory 2).

Further alignment of the DFT structures optimised in the gas phase and the solvent phase gives root mean square deviation (RMSD) values between 0.017Å and 0.207Å (Supplementary Figure 2). The RMSD values between structures under alignment were obtained by applying the `align` function in PyMOL software, which first uses a global dynamic-programming (Needleman–Wunsch[23]) alignment with BLOSUM62 scoring[24] to match atoms between the two structures. All atoms are superimposed by applying a least-squares fitting using the Kabsch algorithm[25] to minimize RMSD value. After this initial alignment, PyMOL performs up to five cycles of iterative refinement, discarding atoms with large deviations (typically beyond ~2 Å or two standard deviations) and repeating the fit until convergence. The final RMSD value after these procedures is then reported. The structures with larger RMSD values (**INT2-4m** at 0.207Å and **TS2a-4m** at 0.200Å) have Gibbs free energy values differing > 2 kcal mol^{-1} (but < 4 kcal mol^{-1}) whereas the structures with small RMSD values have very similar Gibbs free energy values (within 2 kcal mol^{-1}). Overall, the Gibbs free energies between these two methods are similar.

Comparing levels of theory 1 and 3, the use of Pople basis sets with diffuse functions gives ~ 2–3 kcal mol^{-1} lower values for each species. Comparing the single point correction using SMD($CHCl_3$)-M06-2X/def2-TZVP//M06-2X/def2-SVP (level of theory 1) and SMD($CHCl_3$)-DLPNO-CCSD(T)/CBS//M06-2X/def2-SVP (level of theory 4), we see that the intermediates/minima on the PES have almost the same Gibbs free energies, while the TSs for these two theories are within 2 kcal mol^{-1} (no systematic trend). Overall, the choice of either method would be equally valid for the study of the present system. As the Karlsruhe-family basis sets can provide a more well-balanced description for all elements across the periodic table, and the DLPNO-CCSD(T)/CBS is generally considered to be close to the "gold standard" of quantum chemistry CCSD(T)/CBS,[26,27] we applied level of theory 4, SMD($CHCl_3$)-DLPNO-CCSD(T)/CBS//M06-2X/def2-SVP, for all subsequent studies.

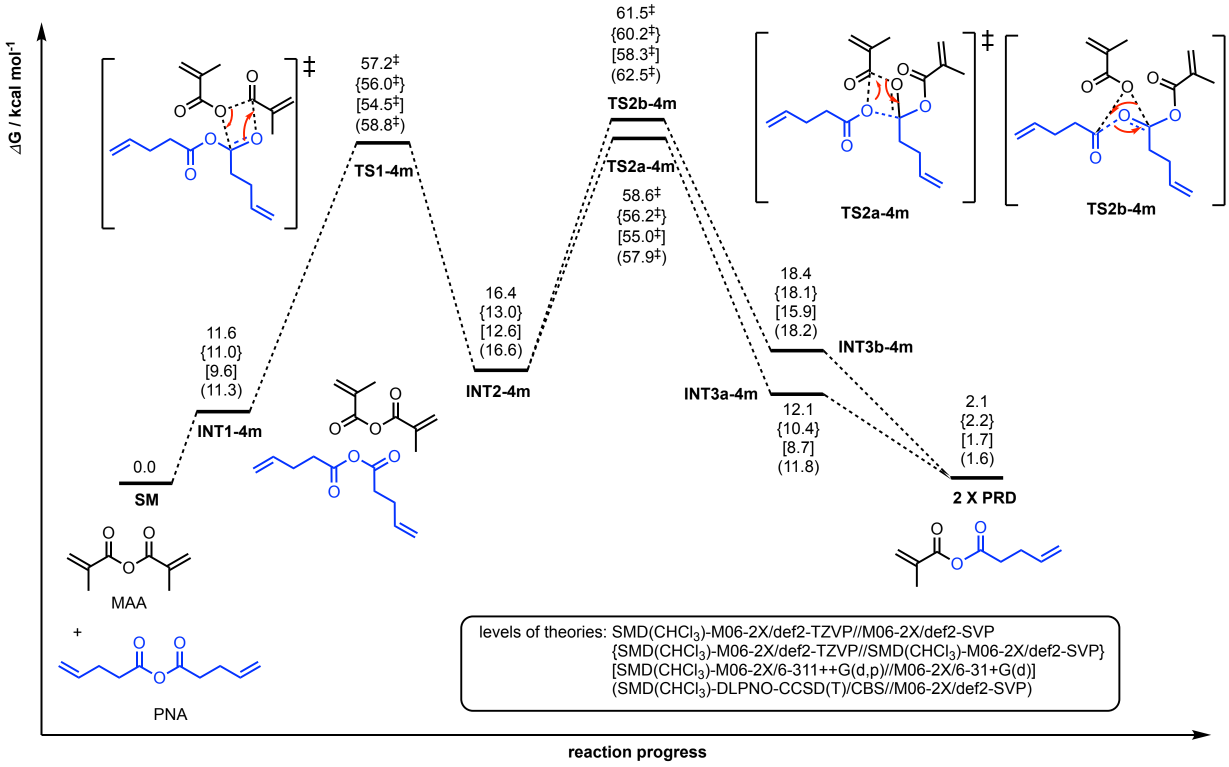

Supplementary Figure 1. Gibbs free energy profile for the dynamic covalent bond exchange via 4-membered transition states (TSs). Four different levels of theories were considered and the values are given for 1) SMD($CHCl_3$)-M06-2X/def2-TZVP//M06-2X/def2-SVP and 2) SMD($CHCl_3$)-M06-2X/def2-TZVP//SMD($CHCl_3$)-M06-2X/def2-SVP in curly braces and 3) SMD($CHCl_3$)-M06-2X/6-311++G(d,p)//M06-2X/6-31+G(d) in square brackets, and 4) SMD($CHCl_3$)-DLPNO-CCSD(T)/CBS//M06-2X/def2-SVP in round brackets. CBS = complete basis set, which results from the extrapolation using double-/triple-ζ cc-pV(DT)Z basis sets.

**PNA**	**MAA**
RMSD = 0.020 Å	0.017 Å
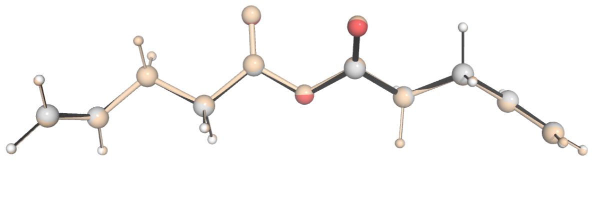	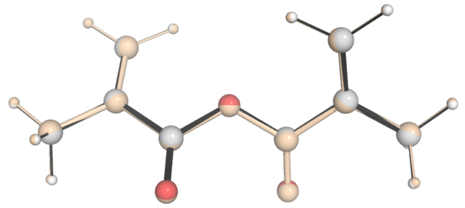
**INT1-4m**	**TS1-4m**
RMSD = 0.073 Å	0.098 Å

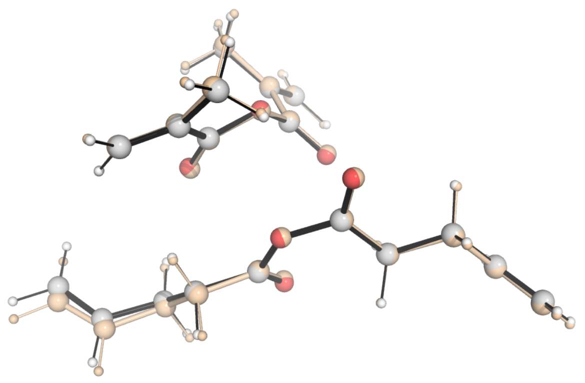	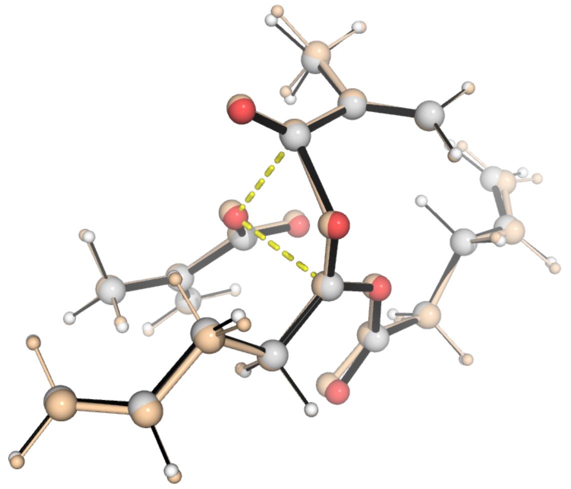
**INT2-4m**	**TS2a-4m**
RMSD = 0.207 Å	0.200 Å
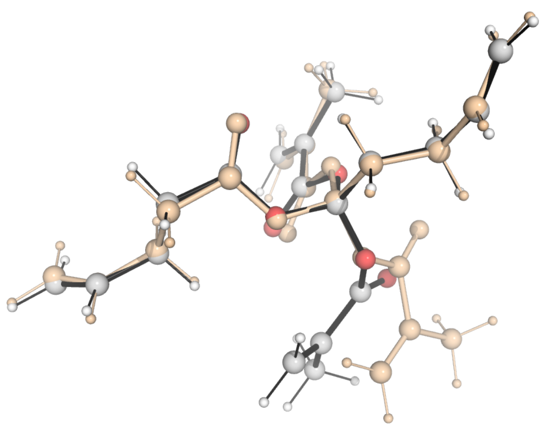	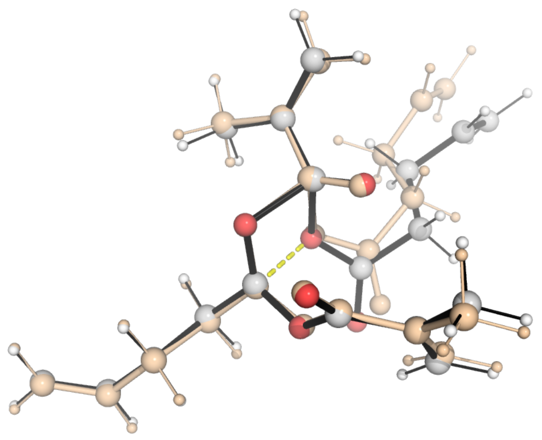
**TS2b-4m**	**INT3a-4m**
RMSD = 0.041 Å	0.081 Å
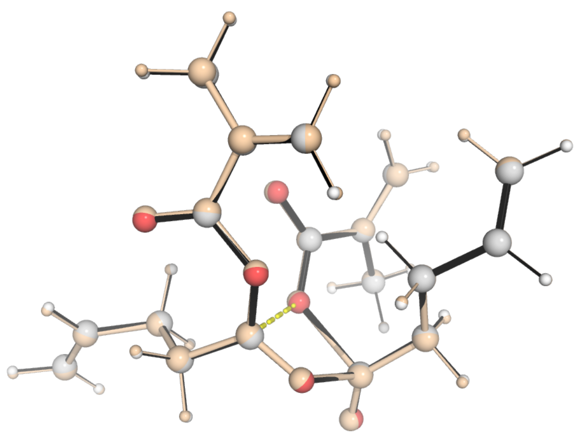	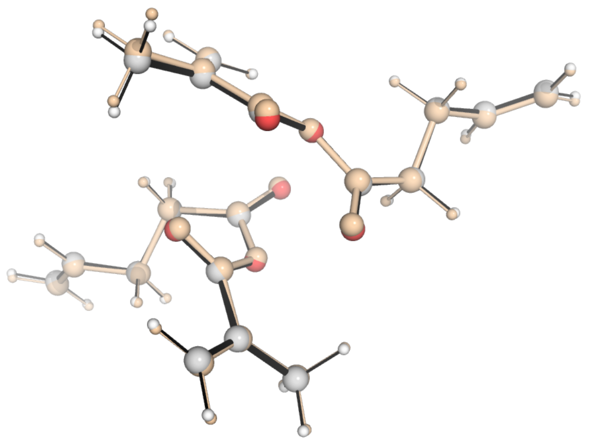

INT3b-4m	PRD
RMSD = 0.064 Å	0.019 Å
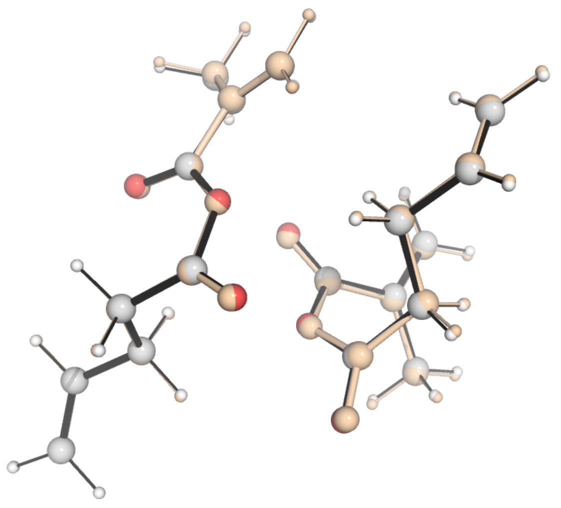	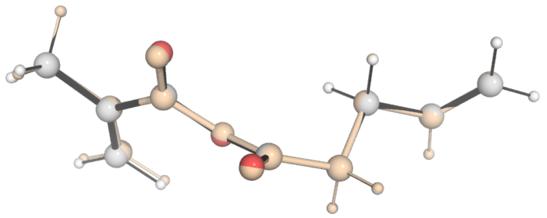

Supplementary Figure 2. Overlay of DFT-optimised structures in gas phase (M06-2X/def2-SVP, normal color) and in solvent phase (SMD($CHCl_3$)-M06-2X/def2-SVP, colored in pale orange) with RMSE value given in Å.

II.2 Concerted mechanism

Firstly, the concerted mechanism was considered (Scheme 2i, pathway a in the main text). An initial "opt=modredundant" constrained TS search with Gaussian gives a good guess structure with desired vibrations, however, upon removing the constraints for a full TS search, no TS structure was located. In addition, the Gaussian *"opt=QST2"* method was applied for the TS search and no positive result was obtained. The ORCA NEB-TS algorithm was used to locate the TS for the concerted mechanism; however, no TS was found.

TS_concerted_6m	TS_concerted_4m
$\Delta G^{\ddagger}$ = 59.3 kcal mol^{-1}	$\Delta G^{\ddagger}$ = 59.3 kcal mol^{-1}
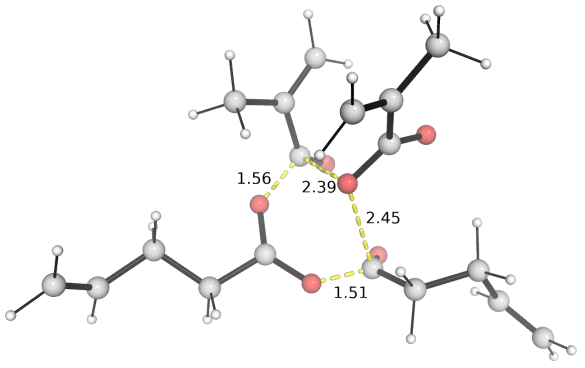	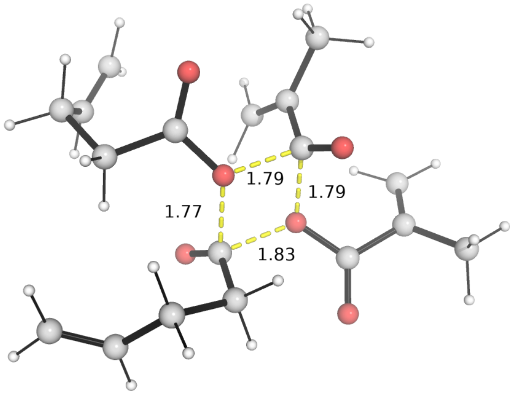

Supplementary Figure 3. DFT optimised structures with key bond distances given in Å.

Upon several trials using the Gaussian *"opt=QST3"* method, we successfully located the TS for the concerted mechanism via 6-membered ring (**TS_concerted_6m**, Supplementary Figure 3). This TS is verified to connect to the desired sides of the reaction by performing IRC analysis (IRC movie in folder *DFT_structures_and_IRC_movie*). Upon solvation correction, this TS gives an activation barrier of 59.3 kcal mol^{-1}. This barrier is much higher than the ones computed for the stepwise mechanism (*vide infra*). An attempt on the conformational sampling of this TS using CREST program was unsuccessful (CREST conformers from this conformational sampling, when used for DFT TS search, did not yield any positive result). We additionally located the concerted mechanism via 4-membered ring (**TS_concerted_4m**, Supplementary Figure 3), which also has a very high barrier, at 59.3 kcal mol^{-1} (IRC movie also attached in folder *DFT_structures_and_IRC_movie*). Therefore, a concerted mechanism for the anhydride bond exchange is ruled out. This high barrier may be due to that the carbonyl group is not activated by, e.g., a Lewis acid or acid coordination to increase its nucleophilicity at carbonyl carbon for nucleophilic attack.

II.3 Relaxed potential energy surface (PES) scan

We considered a relaxed PES scan along the anhydride oxygen atom of MAA and the carbonyl carbon atom of PNA. The scan result is shown in Supplementary Figure 4. As we can see, as the anhydride oxygen atom approaches the carbonyl carbon (shortening O–C distances from structure 1 to structure 2 then to structure 3), the relative energy increases to ~ 60 kcal mol^{-1}. This indicates that there is a large barrier for the formation of O–C bond between the anhydride oxygen atom of MAA and the carbonyl carbon atom of PNA, requiring surmounting a barrier of at least 60 kcal mol^{-1}.

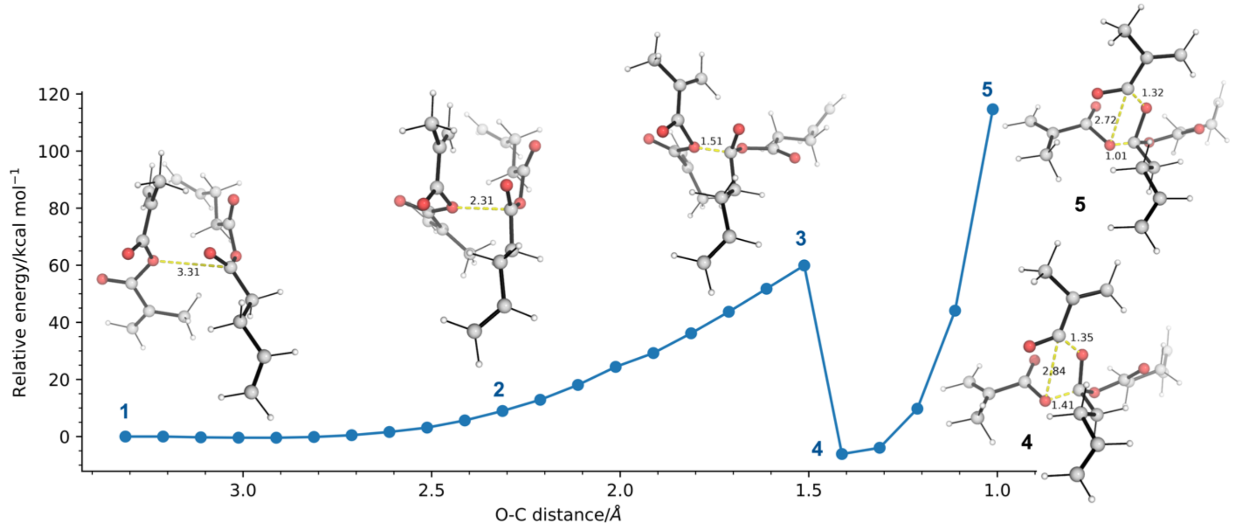

Supplementary Figure 4. Relaxed PES scan energy profile along the O-C distance using M06-2X/def2-SVP level of theory in the gas phase. Gas-phase energy values are used without further corrections.

This barrier will be unfavourable at the reaction conditions. We note that upon going from structure 3 to structure 4 (bond distance shortens from 1.51 Å to 1.41 Å, Supplementary Figure 4), the C–

O bond in the anhydride group of MAA breaks (bond distance of 2.84 Å in structure 4), while the carbonyl carbon forms new C–O bond with the carbonyl oxygen of PNA (bond distance of 1.35 Å in structure 4). This structure (structure 4) is thermodynamically downhill/more stable compared to the reactant complex (structure 1). As the bond distance further shortens to 1.01 Å (structure 5), the energy raises to > 100 kcal mol^{-1}, as the O–C bond is shortened beyond the equilibrium O–C bonding distance.

II.4 Alternative mechanisms

In Supplementary Figure 1, the methacrylic anhydride (MAA) C–O σ-bond is broken in the 4-membered TS structure, **TS1-4m**. We considered the second possibility where the 4-pentenoic anhydride (PNA) σ-bond is broken in a 4-membered TS structure (**TS1'-4m**) and the Gibbs free energy profile is shown in Supplementary Figure 5.

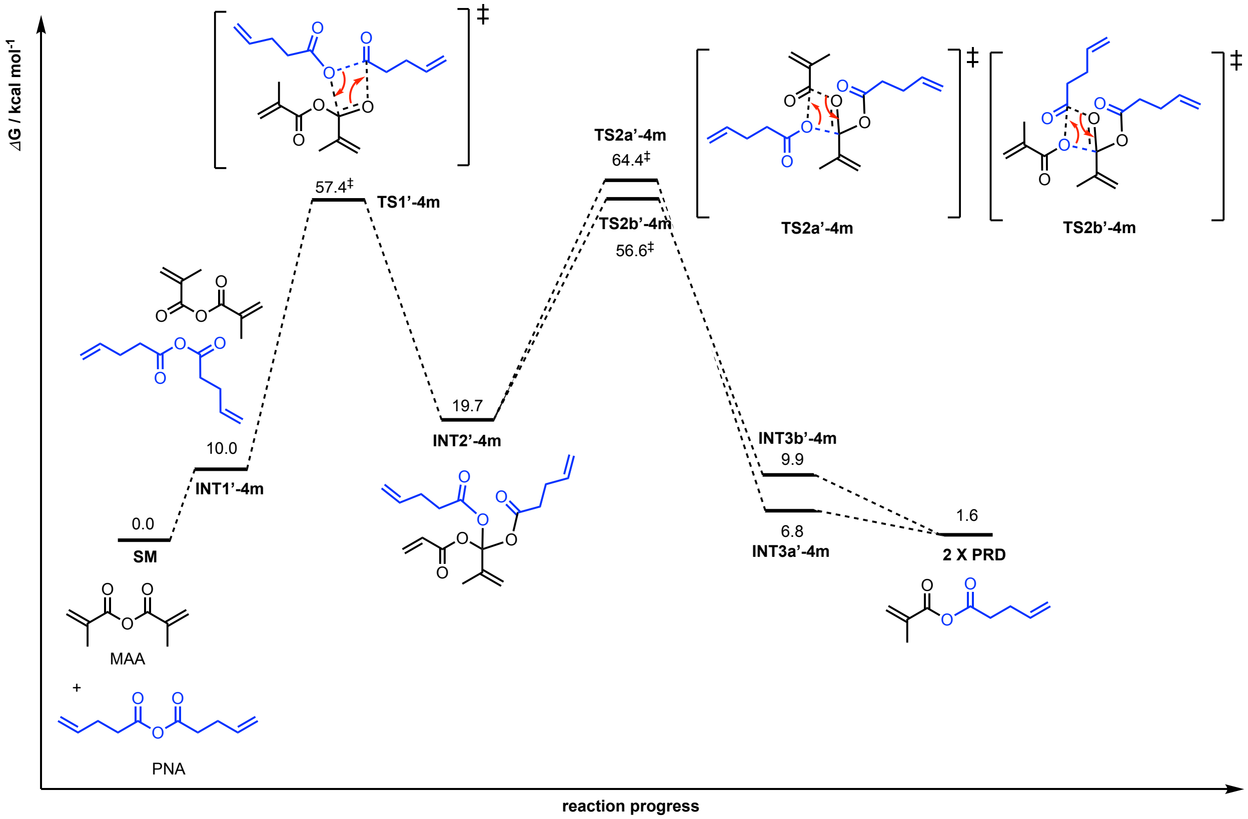

Supplementary Figure 5. Gibbs free energy profile for the dynamic covalent bond exchange via 4-membered TSs, computed at the SMD($CHCl_3$)-DLPNO-CCSD(T)/CBS//M06-2X/def2-SVP level of theory. CBS = complete basis set, which results from the extrapolation using double-/triple-ζ cc-pV(DT)Z basis sets.

The Gibbs free energy profile via the 6-membered TS is shown in Supplementary Figure 6. The activation barrier of the first step was found to be slightly higher than that of the second step. This 6-membered TS, **TS1-6m** has a much lower barrier, at 50.1 kcal mol^{-1}, than **TS1-4m**. The barrier difference of 7.8 kcal mol^{-1} suggests that **TS1-6m** will be kinetically favoured over **TS1-4m** by a factor of > 522,000 : 1, using simple transition state theory (TST) as an estimate at 25ºC.

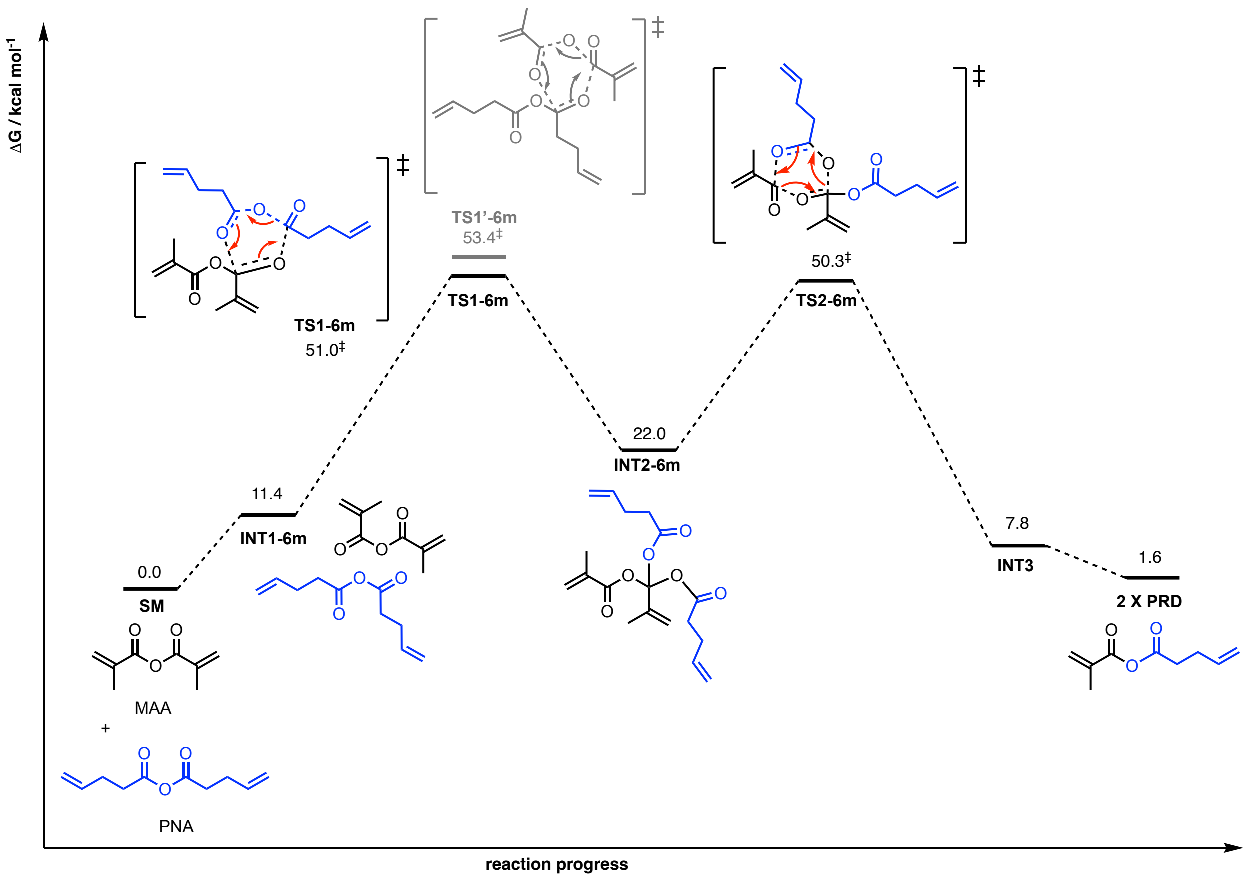

Supplementary Figure 6. Gibbs free energy profile for the dynamic covalent bond exchange via 6-membered TSs, computed at the SMD($CHCl_3$)-DLPNO-CCSD(T)/CBS//M06-2X/def2-SVP level of theory. CBS = complete basis set, which results from the extrapolation using double-/triple-ζ cc-pV(DT)Z basis sets.

II.5 Conformational sampling

We performed thorough conformational sampling to determine each step of the reaction pathway. We do so for the reaction pathways occurring via 6-membered TSs and ignore the pathways via 4-membered TSs, the latter of which are expected to have higher and less favourable activation barrier than the former (compare Supplementary Figures 5 and 6).

II.6 Bond dissociation enthalpies (BDEs)

The bond dissociation enthalpies (BDEs) of the two different anhydride substrates, methacrylic anhydride (MAA) and 4-pentenoic anhydride (PNA) were computed in the gas phase at M06-2X/def2-SVP level of theory. The corresponding radicals were optimised, using unrestricted KS-DFT formalism. The BDE is calculated as

$$\mathrm{BDE(RCOO-COR)} = H_{\mathrm{RCOO\cdot}} + H_{\mathrm{RCO\cdot}} - H_{\mathrm{RCOO-COR}}$$

The BDE values for C–O bond in MAA and PNA are very similar, at 99.8 kcal mol^{-1} for MAA and 99.3 kcal mol^{-1} for PNA.

A potential radical mechanism was considered, by locating the TSs for the diradical formation via the breaking of C–O bond in MAA and PNA. These barriers are found to be 83.3 kcal/mol for MAA and 86.2 kcal/mol for PNA. Thus, potential mechanism via diradical species was ruled out.

II.7 Correlation with experimental data

The kinetics for the anhydride bond exchange using MAA and PNA small molecules as model system have been measured experimentally using ^{13}C NMR spectroscopy in $CDCl_3$ in a heated sealed tube.[22] The relevant kinetic data are shown below:

T/°C	T/K	*k*	1/T	ln(*k*)	ln(*k*/T)
35	308.15	6.92E-07	0.00324517	-14.184	-19.914
40	313.15	1.42E-06	0.00319336	-13.465	-19.212
45	318.15	2.89E-06	0.00314317	-12.754	-18.517
50	323.15	1.77E-05	0.00309454	-10.942	-16.720

Supplementary Table 1. Kinetic parameters measured experimentally using ^{13}C NMR for the anhydride bond exchange between methacrylic anhydride (MAA) and 4-pentenoic anhydride (PNA).

We plot the Arrhenius plot of ln(*k*) against 1/T to obtain the following graph:

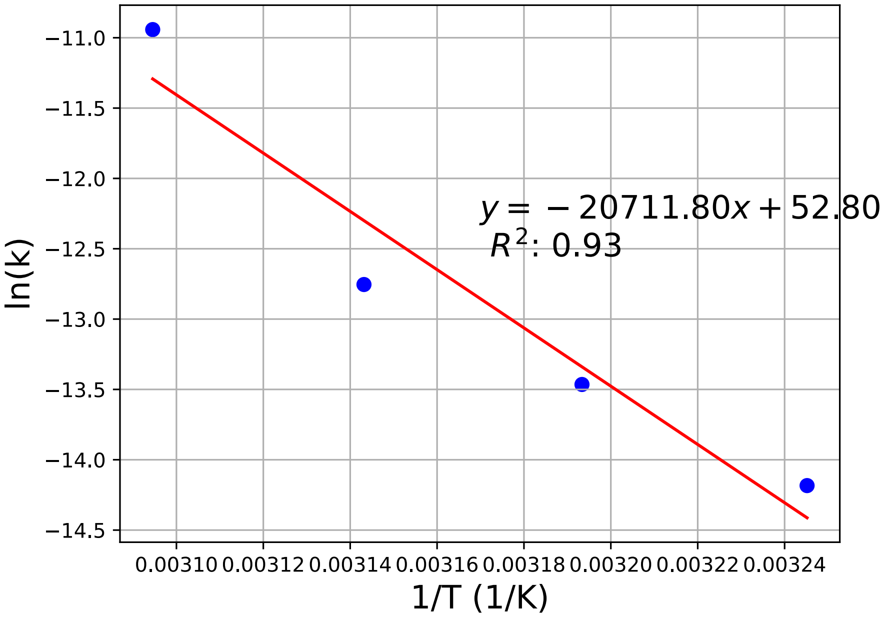

Supplementary Figure 7. Arrhenius plot for the anhydride bond exchange between methacrylic anhydride (MAA) and 4-pentenoic anhydride (PNA).

We can obtain the activation energy from the plot via:

$$\text{y-intercept} = \frac{-E_a}{R} = -20711.80$$
$$\implies E_a = 20711.80 * 8.314 = 41.2 kcal/mol$$

Thus, the enthalpy of activation is given by

$$E_a = \Delta H^{\ddagger} + RT$$
$$\implies \Delta H^{\ddagger} = E_a - RT = 41.2 - 8.314 * 323.15/4184$$
$$= 40.5 \text{ kcal/mol}$$

Using the Eyring-Polanyi plot of ln(*k*/T) against 1/T (Supplementary Figure 8), we obtain the following the entropy of activation as:

$$\text{y-intercept} = \frac{\Delta S^{\ddagger}}{R} + ln\frac{k_B}{h} = 46.05$$
$$\frac{\Delta S^{\ddagger}}{8.314} + ln\frac{1.380649E^{-23}}{6.62607015E^{-34}} = 46.05$$
$$\frac{\Delta S^{\ddagger}}{8.314} = 46.05 - 23.76 = 22.29$$
$$\Delta S^{\ddagger} = 22.29 * 8.314/4184 = 0.04429 \text{ kcal/mol/K}$$

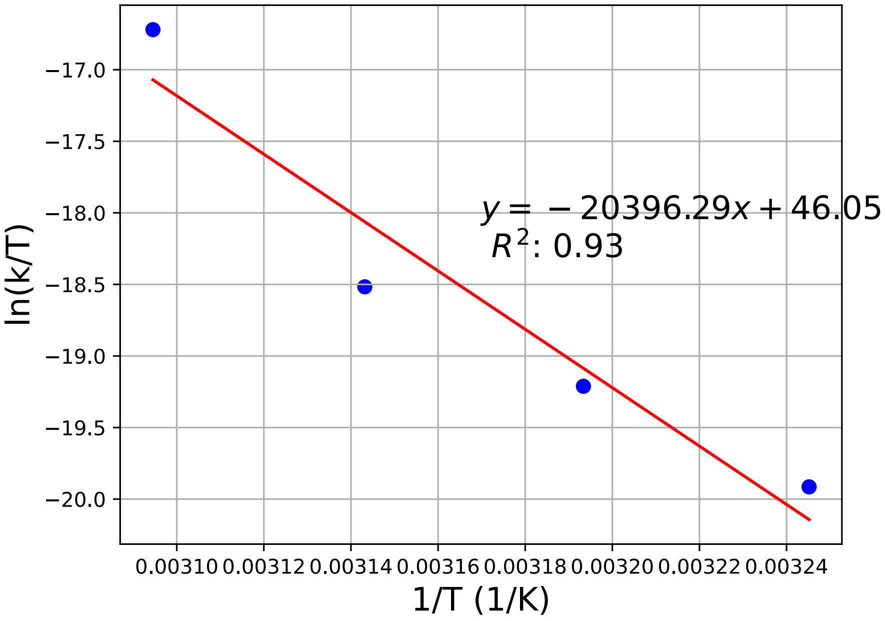

Supplementary Figure 8. Eyring-Polanyi plot for the anhydride bond exchange between methacrylic anhydride (MAA) and 4-pentenoic anhydride (PNA).

Therefore, we can obtain the experimental Gibbs free energy of activation as:

$$\Delta G^{\ddagger} = \Delta H^{\ddagger} - T * \Delta S^{\ddagger}$$
$$= 40.5 - 323.15 * 0.04429$$
$$= 26.2 \text{ kcal/mol}$$

We applied the same method to convert the enthalpy of activation for acid-anhydride bond exchange reported by the Chen group[28] (Supplementary Table 2) into Gibbs free energy.

T/°C	T/K	*k*	1/T	ln(*k*)	ln(*k*/T)
180	453.15	0.000233	0.002207	-8.36544	-14.4817
185	458.15	0.000355	0.002183	-7.94307	-14.0703
195	468.15	0.001093	0.002136	-6.81892	-12.9677
200	473.15	0.002227	0.002113	-6.10702	-12.2664

Supplementary Table 2. Kinetic parameters measured experimentally for the acid-anhydride bond exchange reported by the Chen group.

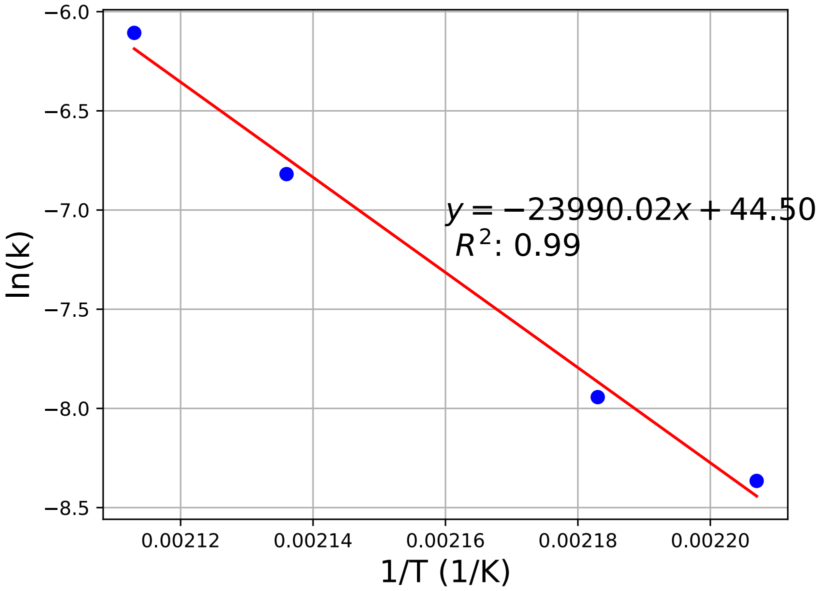

Supplementary Figure 9. Arrhenius plot for the anhydride bond exchange in tri-block copolymer.

We can obtain the activation energy from this plot (Supplementary Figure 9):

$$\text{y-intercept} \ = \frac{-E_a}{R} = -23990.02$$
$$\implies E_a = 23990.02 * 8.314 = 47.7\text{kcal/mol}$$

Thus, the enthalpy of activation is given by

$$E_a = \Delta H^{\ddagger} + RT$$
$$\implies \Delta H^{\ddagger} = E_a - RT = 47.7 - 8.314 * 323.15/4184$$
$$= 47.1\text{kcal/mol}$$

Using the Eyring-Polanyi plot of ln(*k*/T) against 1/T (Supplementary Figure 10), we obtain the following the entropy of activation as:

$$\text{y-intercept} = \frac{\Delta S^{\ddagger}}{R} + \ln\frac{k_B}{h} = 35.39$$

$$\frac{\Delta S^{\ddagger}}{8.314} + \ln\frac{1.380649E^{-23}}{6.62607015E^{-34}} = 35.39$$

$$\frac{\Delta S^{\ddagger}}{8.314} = 35.39 - 23.76 = 11.63$$

$$\Delta S^{\ddagger} = 11.63 * 8.314/4184 = 0.02310989961\text{kcal/mol/K}$$

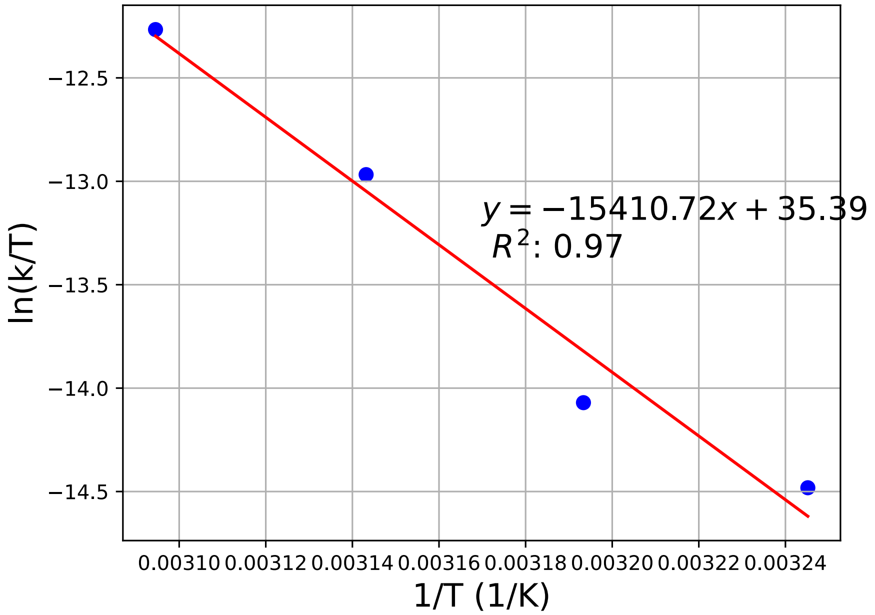

Supplementary Figure 10. Eyring-Polanyi plot for the anhydride bond exchange in tri-block copolymer.

Therefore, we can obtain the experimental Gibbs free energy of activation, at 200°C, as:

$$\begin{aligned}\Delta G^{\ddagger} &= \Delta H^{\ddagger} - T * \Delta S^{\ddagger} \\ &= 47.1 - 473.15 * 0.0231099 \\ &= 36.2\text{kcal/mol}\end{aligned}$$

II.8 Mechanism under acid catalysis

After considering all the mechanistic possibilities, we note that the barriers in the absence of acid catalysis are rather high. The acid-catalysed anhydride exchange mechanism has been studied experimentally by Mironov and Zharkov, whose earlier works showed that anhydride bond exchange can occur readily under acid-catalysis conditions.[22] We performed computational studies to explore this possibility.

We follow the approach for studying the site of protonation by adding a naked proton, as reported by Grimme's work.[29] Since our system has two distinct possible sites for protonation, namely the anhydride carbonyl O atom and the bridging O atom, we investigated the relative stabilities of the

protonated species for each possibility for both MAA and PNA monomer. For both monomers, the starting structure where the proton is near the bridging O atom optimises to the structure where the proton is on one carbonyl O atom (**[MAA-H+]_O2** and **[PNA-H+]_O2**). The optimised DFT structures and their relative Gibbs free energies are given in Supplementary Figure 11.

**[MAA-H+]_O1**	**[MAA-H+]_O2**
$\Delta\Delta G^{\ddagger}$ = 0.0 kcal mol^{-1}	$\Delta\Delta G^{\ddagger}$ = 3.8 kcal mol^{-1}
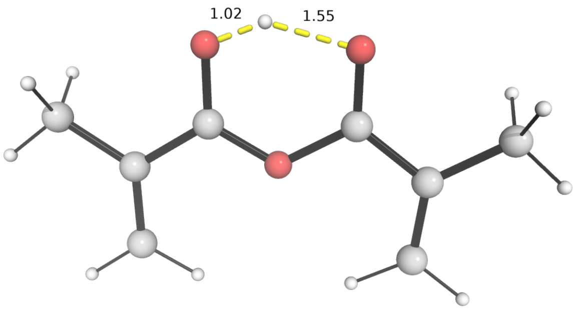	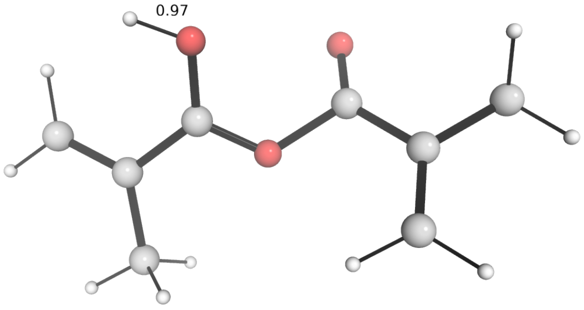
**[PNA-H+]_O1**	**[PNA-H+]_O2**
$\Delta\Delta G^{\ddagger}$ = 1.4 kcal mol^{-1}	$\Delta\Delta G^{\ddagger}$ = 3.6 kcal mol^{-1}
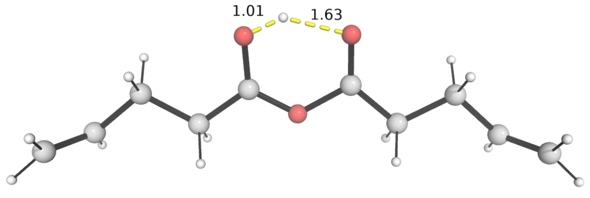	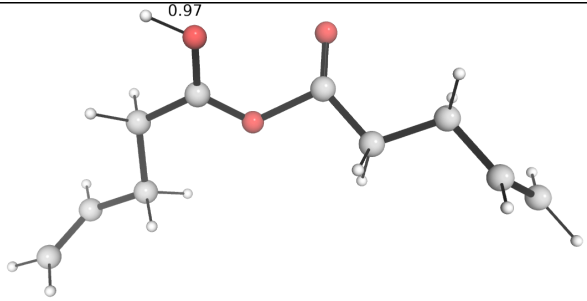

Supplementary Figure 11. DFT optimised structures for the PNA and MAA monomer, with their Gibbs free energies for the protonation process given relative to the most stable structure, **[MAA-H+]_O1**, which forms the proton-bridged 6-member ring.

The TS structure for the cycloaddition via 8-member ring transition state was verified to be the true transition state leading from the reactant complex to the intermediate via IRC analysis (see IRC movie attached). Without conformational sampling, this TS, **TS_acid'**, has a barrier of 30.0 kcal/mol, which upon further conformational sampling and DFT TS optimization, reduced to 25.9 kcal/mol, **TS_acid**, in Supplementary Figure 12.

**TS_acid**	**TS_acid'**
$\Delta G^{\ddagger}$ = 25.9 kcal mol^{-1}	$\Delta G^{\ddagger}$ = 30.0 kcal mol^{-1}

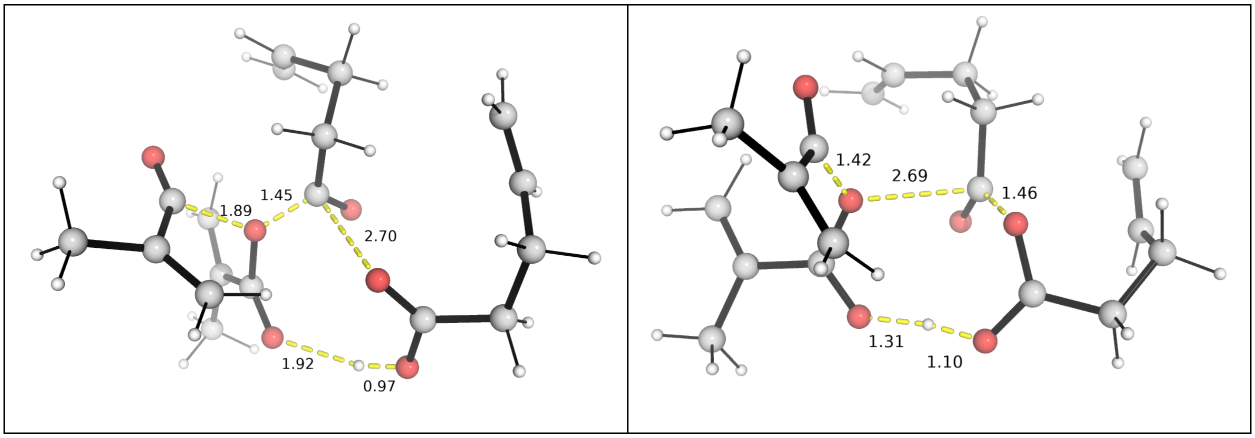

Supplementary Figure 12. DFT optimised structures for anhydride bond exchange under acid calaysis, with key bond distances given in Å. Gibbs free energies are given with respect to the reactant complex, **RCT_acid**.

II.9 Effect of Temperature on Gibbs free energy correction

The Gibbs free energy profile has been computed with temperature correction at 25ºC to check against the experimentally obtained barrier using 13C NMR spectroscopy in CDCl3.[22] To assess the effect of temperature change on the activation barriers and the Gibbs free energy profile, we recomputed Gibbs free energy profile for the uncatalysed and acid-catalysed reaction in different temperatures (50ºC, 100ºC, 150ºC, 180ºC, 200ºC). The results are summarised in Tables S3–5 below.

We see that for uncatalysed system, the dynamic covalent bond exchange mechanism via 6-membered ring transition states involving the breaking of C–O bond in **MAA** (Supplementary Table 3) is still favoured over breaking of C–O bond in **PNA** (Supplementary Table 4), at all temperatures, such that the preferred pathway will proceed via Scheme 3a, main text. The overall barrier ranges from 44.1 kcal/mol at 25ºC, to 52.8 kcal/mol at 200ºC for uncatalysed reaction, although the barrier from **INT1** to **TS1** stay in the range of 35.1 kcal/mol at 25ºC to 36.1 kcal/mol at 200ºC (Supplementary Table 3). The increase in the barrier can be rationalised as such: as temperature increases, the molecules will move with a higher kinetic energy, making the formation of (reactant and TS) complex more difficult. Further analysis using the energies of (**TS1** – **MAA** – **PNA**) shows that in this process, the $\Delta H^{\ddagger}$ component of $\Delta G^{\ddagger} = \Delta H^{\ddagger} - T\Delta S^{\ddagger}$ remains similar across different temperatures (28.4 kcal/mol at 25ºC to 28.6 kcal/mol at 200ºC) whereas the $-T\Delta S^{\ddagger}$ term increases from 15.3 kcal/mol at 25ºC to 23.7 kcal/mol at 200ºC, without quasi-RRHO correction and from 15.6 kcal/mol at 25ºC to 24.2 kcal/mol at 200ºC, with quasi-RRHO correction. Thus, the temperature increase mostly affects the term $-T\Delta S^{\ddagger}$, due to its temperature contribution.

For the acid catalysed reaction, a similar trend is observed: the barrier increases from 25.6 kcal/mol at 25ºC to 33.2 kcal/mol at 200ºC (Supplementary Table 5), although the barriers from **INT1_acid** to **TS1_acid** remain in a narrow range (24.1 kcal/mol at 25ºC to 24.2 kcal/mol at 200ºC, Supplementary Table 5).

T/^{o}C	INT1	TS1	INT2	TS2a	TS2b	INT3	PRD	TS1 – INT1
25	9.0	**44.1**	9.9	42.4	44.3	7.8	0.7	35.1
50	10.1	**45.3**	11.1	43.7	44.2	8.9	0.8	35.2
100	12.4	**47.8**	13.4	46.2	46.8	11.0	0.9	35.4
150	14.6	**50.3**	16.0	48.8	49.4	13.1	1.1	35.7
180	15.9	**51.8**	17.5	50.3	50.9	14.3	1.1	35.9
200	16.7	**52.8**	18.5	51.3	52.0	15.2	1.2	36.1

Supplementary Table 3. Gibbs free energies for species in the dynamic covalent bond exchange mechanism via 6-membered ring transition states involving the breaking of C–O bond in methacrylic anhydride (MAA), corresponding to Figure 2a in the main text. Values in bold indicate rate-determining step barrier.

T/^{o}C	INT1	TS1'	INT2'	TS2a'	TS2b'	INT3	PRD	TS1'– INT1
25	9.0	**45.2**	12.3	44.2	46.2	7.8	0.7	36.2
50	10.1	**46.5**	13.5	45.5	47.4	8.9	0.8	36.4
100	12.4	**49.1**	16.0	48.0	49.9	11.0	0.9	36.7
150	14.6	**51.7**	18.5	50.5	52.3	13.1	1.1	37.1
180	15.9	**53.2**	19.9	52.0	53.8	14.3	1.1	37.3
200	16.7	**54.2**	20.9	53.0	54.8	15.2	1.2	37.5

Supplementary Table 4. Gibbs free energies for species in the dynamic covalent bond exchange mechanism via 6-membered ring transition states involving the breaking of C–O bond in 4-pentenoic anhydride (PNA), corresponding to Figure 2b in the main text. Values in bold indicate rate-determining step barrier.

T/^{o}C	INT1_acid	TS1_acid	INT2_acid	PRD_acid	TS1_acid – INT1_acid
25	1.8	**25.9**	14.8	2.7	24.1
50	2.8	**26.9**	15.7	2.7	24.1
100	4.8	**29.0**	17.5	2.8	24.2
150	6.8	**30.1**	19.3	2.9	23.3

180	8.0	**32.2**	20.4	3.0	24.2
200	8.8	**33.0**	21.1	3.0	24.2

Supplementary Table 5. Gibbs free energies for species in the acid-catalysed dynamic covalent bond exchange mechanism. Values in bold indicate rate-determining step barrier.

II.10 Effect of inclusion of D3 correction in M06-2X in resulting Gibbs free energy profile

As suggested by one reviewer, we performed additional studies on the major pathway using M06-2X-D3/def2-SVP for geometry optimisation, followed by DLPNO-CCSD(T) single point solvent correction to ascertain the effect of inclusion of D3 correction on the Gibbs free energy profile. We took the lowest energy conformers of PES for the major pathway and performed additional studies by reoptimising the structures using M06-2X-D3, followed by SP DLPNO-CCSD(T) with solvent correction. The following show the comparison of energy profiles, with the SMD(chloroform)-DLPNO-CCSD(T)//M06-2X-D3/def2-SVP results in square brackets and originally computed SMD(chloroform)-DLPNO-CCSD(T)// M06-2X/def2- SVP shown. We observed that the energy profile thus obtained (Supplementary Figure 13) is no different either with D3 correction or not, when using M06-2X functional, in agreement with other studies.

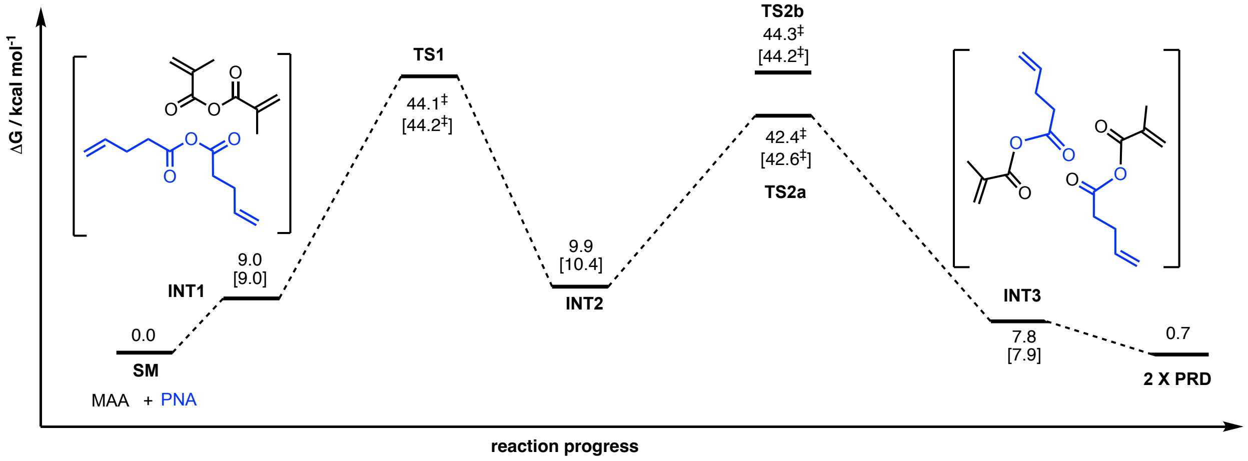

Supplementary Figure 13. Gibbs free energy profile for the dynamic covalent bond exchange via 6-membered TSs via Pathway I shown in Scheme 3a) in the main text. Shown are energies from SMD(chloroform)-DLPNO-CCSD(T)// M06-2X[-D3]/def2-SVP where the values in square brackets are results from the inclusion of D3 correction.

II.11 Effect of Boltzmann weighting in resulting Gibbs free energy profile

To access the effect of Boltzmann weighting on the potential energy surface, we performed Boltzmann weighting on the dynamic covalent bond exchange via 6-membered TSs via Pathway I shown in Scheme 3a) in the main text. The resulting comparison is shown in Supplementary Figure 14. We note a small change in the values for each species, but the overall conclusion that TS1 is the rate-determining step remains valid. For simplicity, we adopt the PES discussion without Boltzmann weighting but using the most stable conformer throughout, although we note

that, complete/adequate conformational sampling is required to ascertain the main rate-determining step.

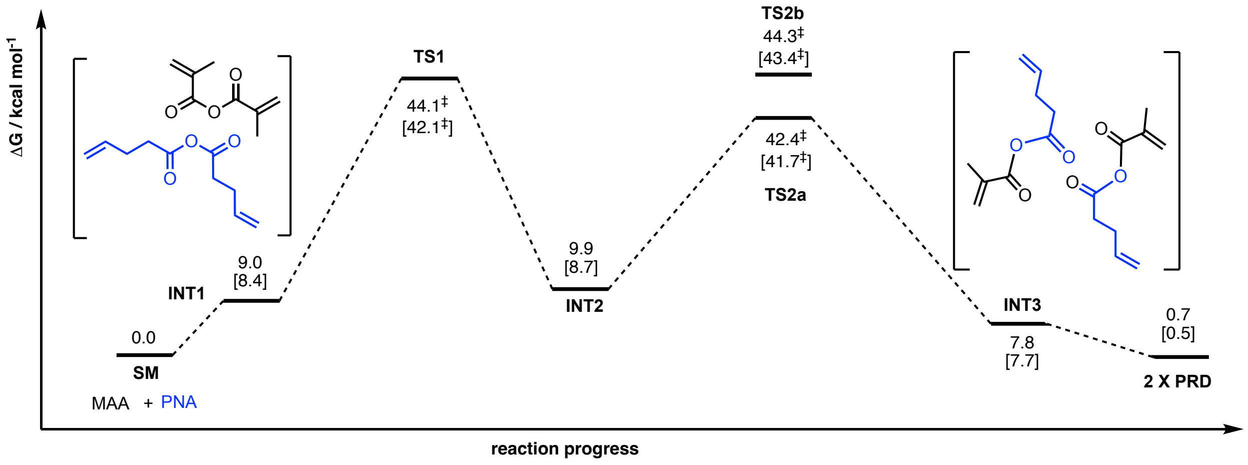

Supplementary Figure 14. Gibbs free energy profile for the dynamic covalent bond exchange via 6-membered TSs via Pathway I shown in Scheme 3a) in the main text. Shown are energies from SMD(chloroform)-DLPNO-CCSD(T)// M06-2X/def2-SVP where the values in square brackets are results from Boltzmann weighting.

II.12 Frontier molecular orbitals, natural bond orbital (NBO) charges, non-covalent interaction (NCI) plots and distortion-interaction/activation-strain analyses

To understand the molecular origins governing the transition state barrier heights, we performed frontier molecular orbitals (HOMO and LUMO plots), natural bond orbital (NBO) charges and distortion-interaction/activation-strain analyses for key transition states.

The NBO charges of the key atoms in the TSs, the HOMO and LUMO plots for the key TSs for both uncatalysed and catalysed pathways are shown in Supplementary Figure 15. The NBO charges analyses show that all the O atoms in the TSs for the uncatalysed pathways have similar charges at corresponding positions; these O atoms have NBO charges fall within a relative narrow range from -0.587 a.u. to -0.732 a.u.. In each TS for the uncatalysed pathways, the O atoms having the least negative and most negative NBO charges form part of the 6-membered ring in the TS structure (Supplementary Figure 15). For carbon atoms attached to the O atoms, corresponding carbon atoms in these TSs also have similar values – these are positively charged and with NBO charges in the range from 0.873 a.u. to 0.983 a.u.. These results indicate a concerted mechanism where the electrons flow from O to C, consistent with a nucleophilic attack on carbon. The consistent NBO charges among TSs indicate a similar electronic environment in all uncatalysed TS structures, suggesting that they proceed via similar mechanistic pathways.

For the catalysed pathway, transition state, **TS1_acid**, has O atom that has a much less negative NBO charge, such as one that is -0.369 a.u.. This is because this O atom is part of the fragment that forms the acylium ion (R–C≡O$^+$), that has electrons donation to carbon atom (This species is subsequently attacked by a carboxylate to form the mixed anhydride). The greater charge distribution of the O atoms in the acid-catalysed pathway, as evidenced by larger differences in

charges (e.g., from -0.369 to -0.712 a.u.), also indicates a protonation-induced activation of the electrophilic site and better charge separation facilitated by the catalyst.

<table>
<tr><th></th><th>NBO Charges</th><th>HOMO</th><th>LUMO</th></tr>
<tr><td>TS1</td><td>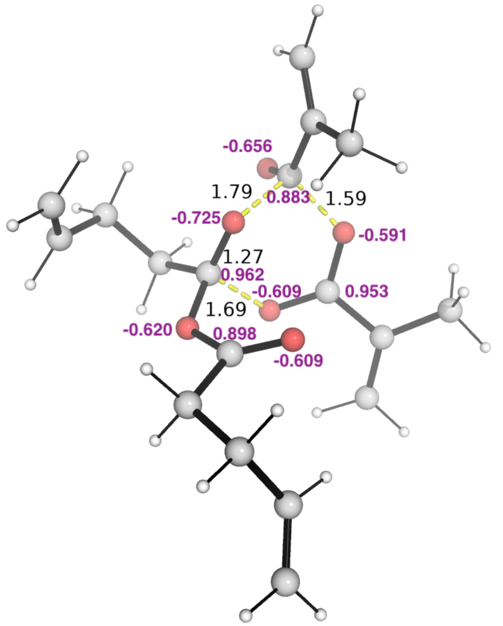
</td><td>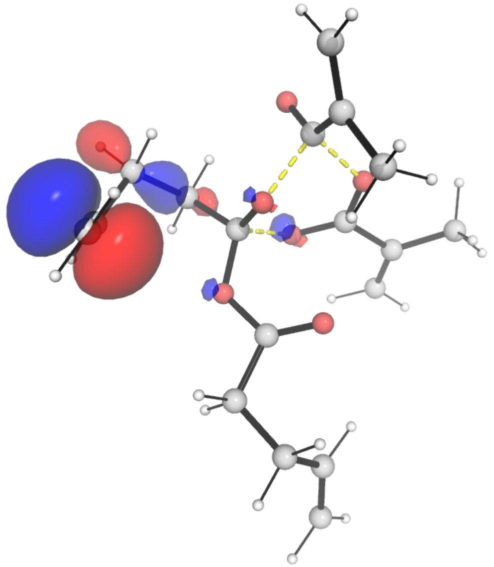</td><td>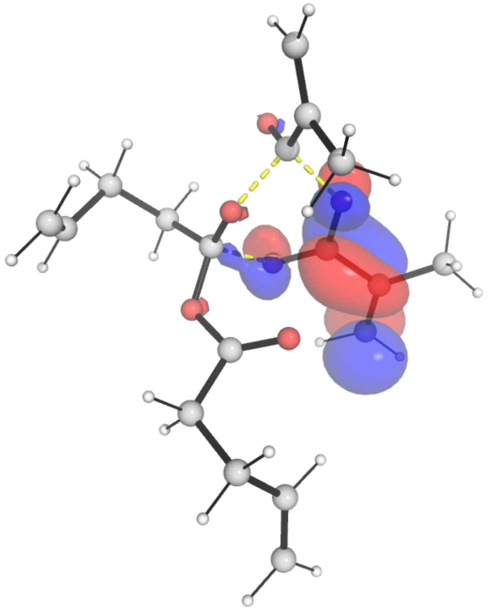</td></tr>
<tr><td>TS2a</td><td>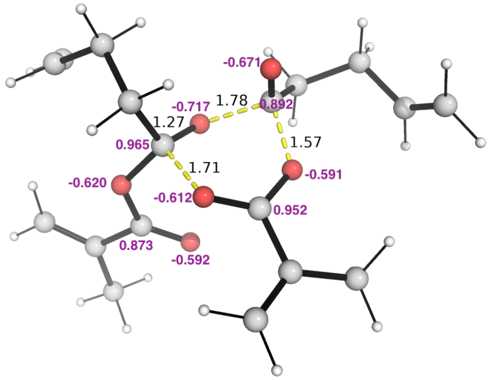
</td><td>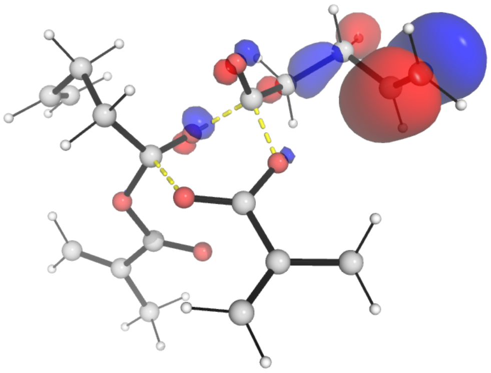</td><td>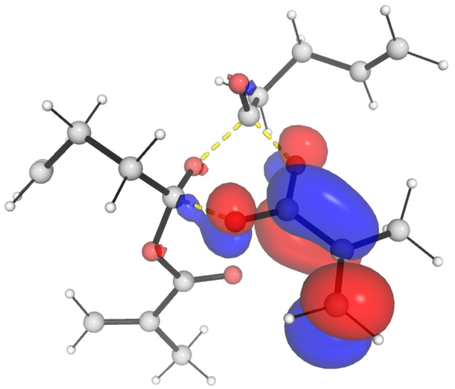</td></tr>
</table>

**TS2b**	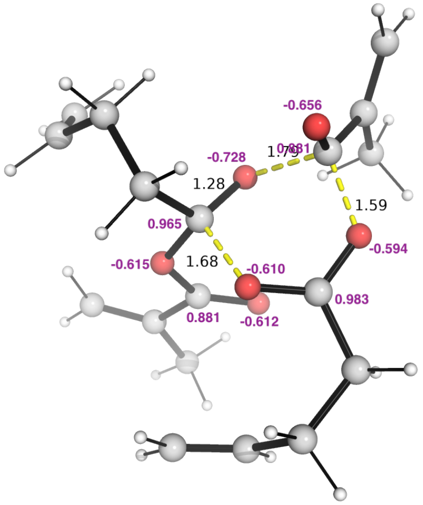 	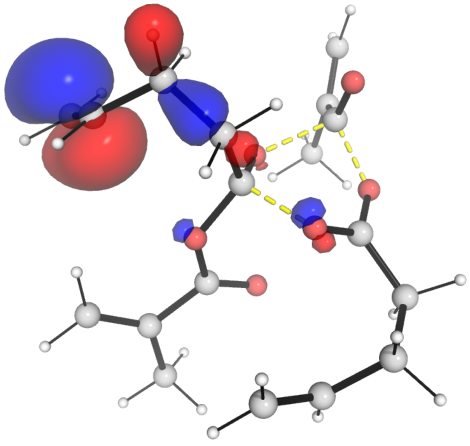	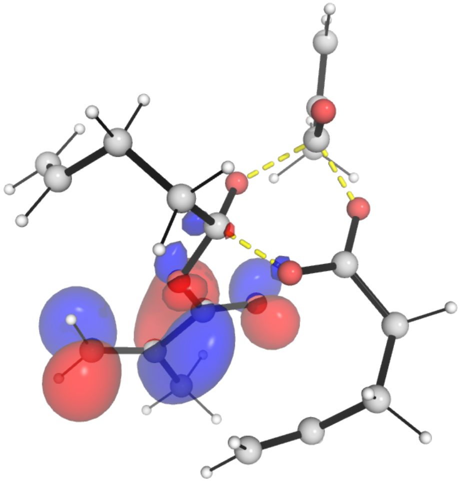
**TS1'**	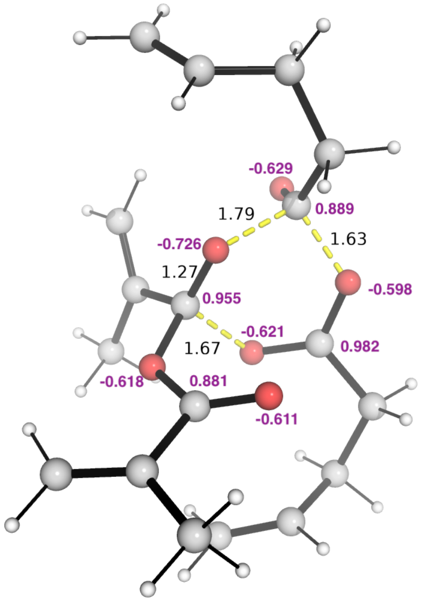 	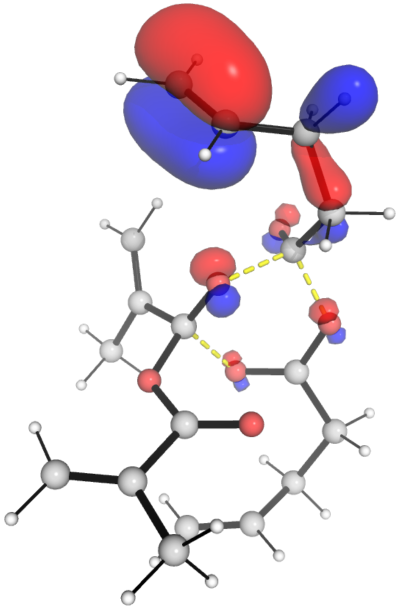	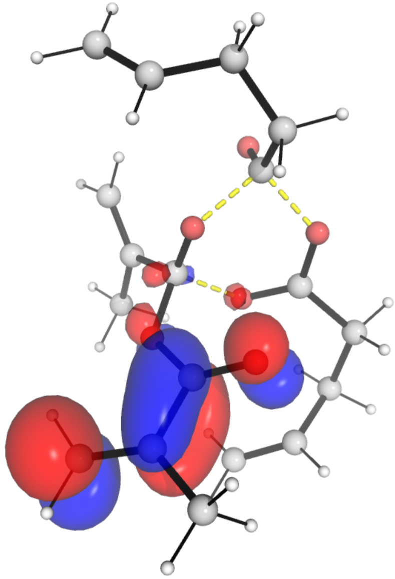

<table>
<tr>
<td>TS2a’</td>
<td>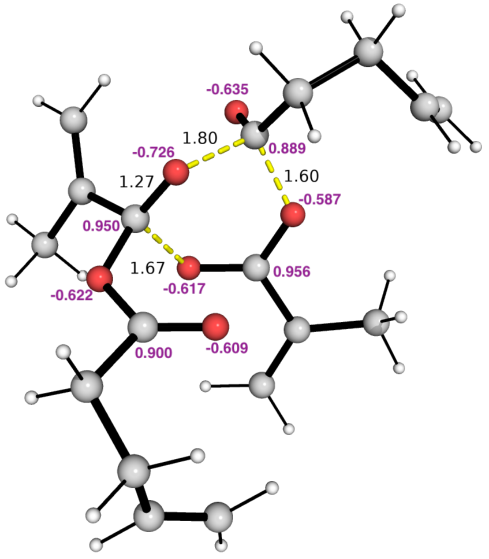
</td>
<td>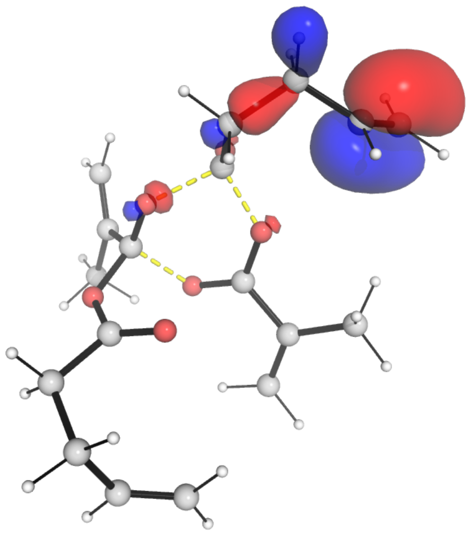</td>
<td>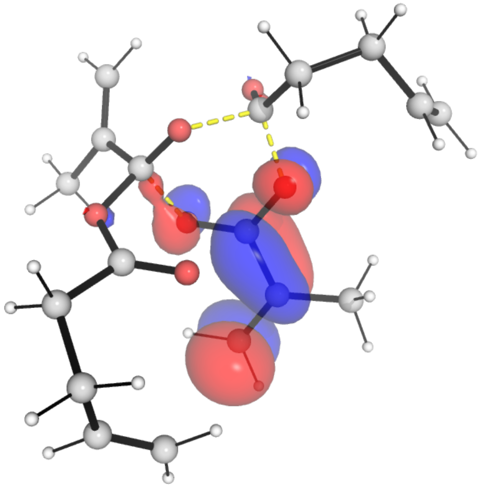</td>
</tr>
<tr>
<td>TS2b’</td>
<td>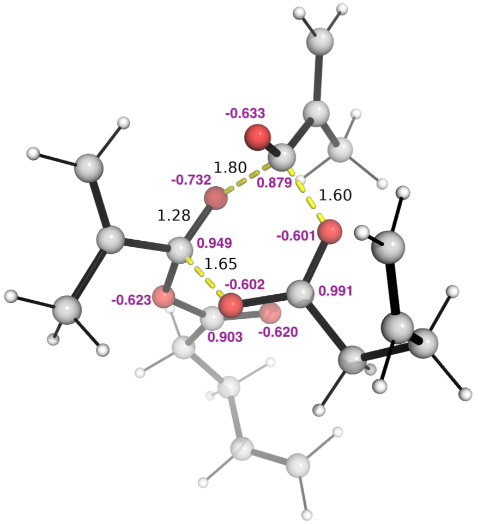
</td>
<td>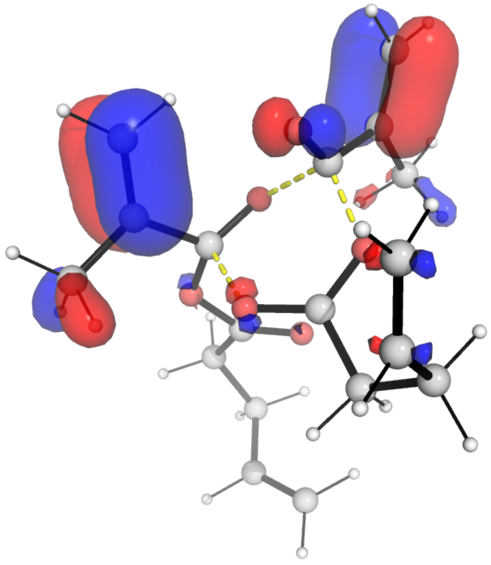</td>
<td>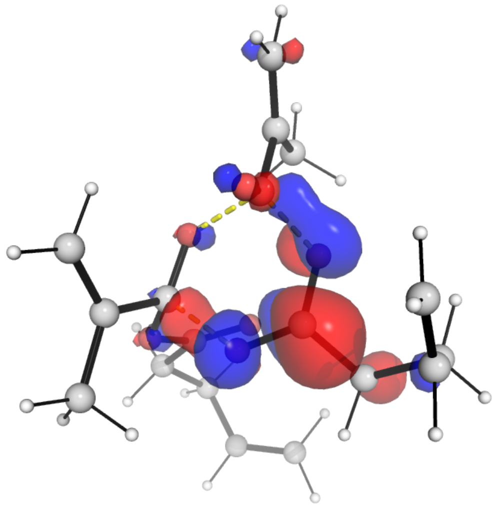</td>
</tr>
</table>

TS1_acid

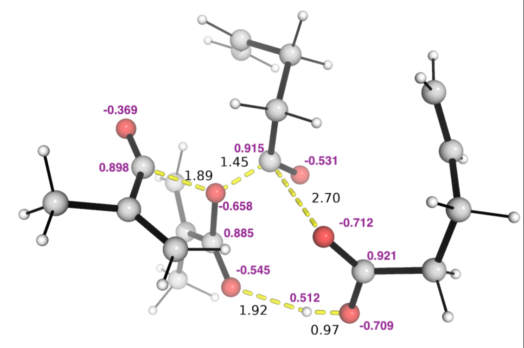

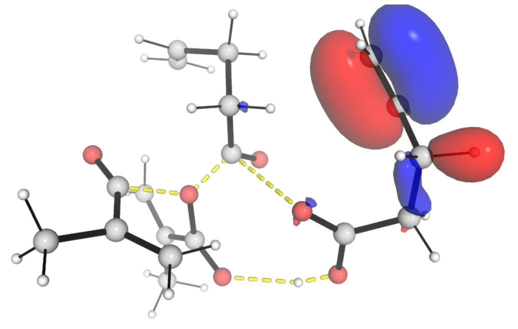

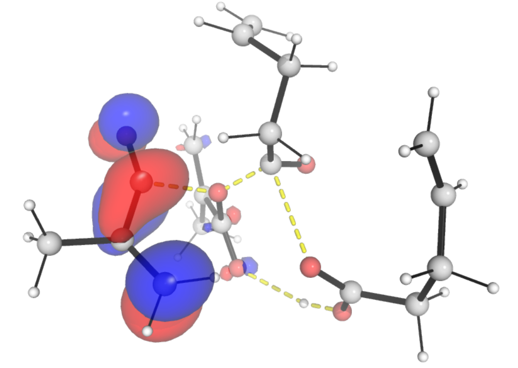

Supplementary Figure 15. Natural bonding orbital (NBO) charges for key atoms and frontier molecular orbital (FMO) plots including HOMO and LUMO plots for the lowest energy TSs for both uncatalysed and catalysed reaction pathways.

Analysis of the FMO plots indicates that the FMO structures are similar, with the notable exception in **TS2b'**. However, despite this noted difference, both the HOMO and LUMO have dominant coefficients on the peripheral of the reacting species and not form core of the 6-membered transition state ring (Supplementary Figure 15). Notably, the HOMO is largely localised on the electron-rich π bonds on C=C side group of the reacting monomers, which may suggest that nucleophilic attack or electron donation originates from regions outside the ring, thus helping to initiate and/or stabilise the TS. For the LUMO, it is mostly localised on the π* systems of carboxylate group, implying that electrophilic activation occurs peripherally, and the 6-membered ring TS forms as a consequence of these interactions. These analysis may also suggest that the atoms within the 6-membered ring TS may be electronically neutral.

Distortion-interaction[30–32] analysis is applied to key TSs to discern the factors affecting the barriers of the lowest energy transition states in each mechanistic pathway. The transition state structures are decomposed by dividing the PNA and the MAA monomers as components. For the acid catalysed transition state, TS1_acid, the two components are the neutral PNA and the protonated MAA, since the protonation of MAA is more favoured than the protonation of PNA (Supplementary Figure 11). Single point calculations with SMD(chloroform) solvent correction were applied performed at DLPNO-CCSD(T)/CBS level of theory to obtain distortion and interaction energies. The distortion energy is given by:

$$E_{dist} = E_{TS,frag1} + E_{TS,frag2} - (E_{eq,frag1} + E_{eq,frag2})$$

where *TS,frag1,2* represent individual fragments in their distorted transition state geometries; and *eq,frag1,2* represent individual fragments in their optimized, equilibrium ground-state geometries; the interaction energy is given by:

$$E_{int} = E_{TS} - (E_{TS,frag1} + E_{TS,frag2})$$

which accounts for the stabilizing interactions (e.g., electrostatic, orbital, dispersion) between the distorted fragments in the TS.

Thus, the total activation energy is given by:

$$\Delta E^{\ddagger} = E_{dist} + E_{int.}$$

Note that this single point activation energy and the activation energy differences $\Delta\Delta E^{\ddagger}$ between the TSs (**Supplementary Table 6**) may be different from the Gibbs free energy differences $\Delta\Delta G^{\ddagger}$

that is computed fully (including vibrational frequencies analysis) at SMD(chloroform)-DLPNO-CCSD(T)/CBS//M06-2X/def2-SVP level of theory.

Transition State	$\Delta E^{\ddagger}$	E_{dist}	E_{int}
**TS1**	28.1	43.6	-15.5
**TS2a**	26.2	171.5	-145.3
**TS2b**	27.5	164.1	-136.6
**TS1'**	28.6	49.0	-20.4
**TS2a'**	28.1	158.1	-130.0
**TS2b'**	30.4	180.4	-150.0
**TS1_acid**	13.5	232.0	-218.5

Supplementary Table 6. Distortion-interaction analysis applied to key transition states.

We note that this decomposition of TSs by dividing the PNA and the MAA monomers as components may work well for **TS1** and **TS1'** but not necessarily for **TS2a**, **TS2b**, **TS2a'**, **TS2b'**, since the anhydride monomer may have already undergone substantial geometry changes upon reaching the second TS. Nevertheless, we note that overall, the first step is rate-determining and that the high activation barriers arises predominantly due to the strain energies experienced by the reacting components when they reorganise in the 6-membered ring transition states.

III. DFT-optimised structures and absolute energies

Geometries of all optimised structures (in *.xyz* format with their associated energy in Hartrees) are included in a separate folder named *DFT_structures_and_IRC_movie* with an associated readme.txt file. All these data have been deposited with this Supplementary Information and uploaded to https://zenodo.org/records/16625442 (DOI: 10.5281/zenodo.16625442).

Supplementary Table 7. Absolute values (in Hartrees) for SCF energy, zero-point vibrational energy (ZPE), enthalpy and quasi-harmonic Gibbs free energy (at 25°C/298.15 K) for optimised structures are given below. Single point corrections in SMD Chloroform for the reaction studies or in C-PCM environments (varying dielectric constants) are also included.

**Mechanistic studies for MAA + PNA anhydride bond exchange in gas phase**						
**Structure**	**E/au**	**ZPE/au**	**H/au**	**T.S/au**	**qh-G/au**	**SP DLPNO-CCSD(T)/CBS**
**MAA_c1**	-535.89586	0.167081	-535.71583	0.049232	-535.763003	-535.9324285

**MAA_c2**	-535.89439	0.167034	-535.71438	0.049479	-535.761613	-535.9321499
**MAA_c3**	-535.8936	0.167027	-535.71361	0.049549	-535.76081	-535.9314652
**MAA_c4**	-535.89458	0.167029	-535.71462	0.049292	-535.761752	-535.9309761
**MAA_c5**	-535.89458	0.16703	-535.71462	0.049291	-535.761751	-535.930976
**MAA_c6**	-535.89458	0.16703	-535.71462	0.049291	-535.761751	-535.930976
**MAA_c7**	-535.89458	0.167031	-535.71462	0.049289	-535.761749	-535.9309758
**MAA_c8**	-535.89458	0.167032	-535.71462	0.049287	-535.761747	-535.9309738
**MAA_c9**	-535.89345	0.167198	-535.7134	0.048927	-535.760242	-535.9298509
**MAA_c10**	-535.89345	0.167199	-535.7134	0.048923	-535.760239	-535.9298491
**MAA_c11**	-535.89347	0.166944	-535.7136	0.049391	-535.760744	-535.9292552
**MAA_c12**	-535.89296	0.167162	-535.71298	0.048819	-535.759725	-535.9292984
**MAA_c13**	-535.889	0.167235	-535.70906	0.047743	-535.755372	-535.9267171
**MAA_c14**	-535.8857	0.167331	-535.70577	0.047509	-535.751852	-535.9227985
**PNA_c1**	-614.40942	0.223918	-614.16965	0.058153	-614.22399	-614.4499867
**PNA_c2**	-614.41095	0.224743	-614.17089	0.055637	-614.22353	-614.4509797
**PNA_c3**	-614.41095	0.224743	-614.17089	0.055637	-614.22353	-614.4509794
**PNA_c4**	-614.41095	0.224743	-614.17089	0.055635	-614.223529	-614.4509789
**PNA_c5**	-614.41023	0.224985	-614.16997	0.055224	-614.222383	-614.4509257
**PNA_c6**	-614.41023	0.224985	-614.16997	0.055214	-614.22238	-614.4509218
**PNA_c7**	-614.41023	0.224986	-614.16997	0.055213	-614.222379	-614.4509217
**PNA_c8**	-614.41023	0.224986	-614.16998	0.055207	-614.222377	-614.4509226
**PNA_c9**	-614.41023	0.224988	-614.16997	0.05521	-614.222377	-614.4509207
**PNA_c10**	-614.41023	0.224988	-614.16997	0.055208	-614.222376	-614.4509194
**PNA_c11**	-614.41055	0.224572	-614.17055	0.056751	-614.223788	-614.4497136
**PNA_c12**	-614.41144	0.224793	-614.1714	0.055381	-614.22398	-614.4502281
**PNA_c13**	-614.41086	0.224617	-614.17094	0.055386	-614.223477	-614.4500614
**PNA_c14**	-614.41086	0.224621	-614.17093	0.055383	-614.223472	-614.4500605
**PNA_c15**	-614.41086	0.224621	-614.17093	0.055378	-614.22347	-614.4500609
**PNA_c16**	-614.41086	0.22462	-614.17093	0.055379	-614.223471	-614.4500584
**PNA_c17**	-614.41086	0.22462	-614.17093	0.05538	-614.223471	-614.4500581
**PNA_c18**	-614.41124	0.225072	-614.17089	0.056178	-614.223719	-614.4493537
**PNA_c19**	-614.41124	0.225073	-614.17089	0.056161	-614.223711	-614.4493525
**PNA_c20**	-614.40875	0.225214	-614.16822	0.056273	-614.221032	-614.449153

**INT1**	-1150.3323	0.393124	-1149.9106	0.086065	-1149.990279	-1150.391776
**INT1_c2**	-1150.333	0.393272	-1149.9111	0.086369	-1149.99099	-1150.391564
**INT1_c3**	-1150.3311	0.393767	-1149.9088	0.086285	-1149.988375	-1150.391147
**INT1_c4**	-1150.3331	0.393806	-1149.9109	0.085246	-1149.990118	-1150.390578
**INT1_c5**	-1150.3332	0.394064	-1149.9109	0.084557	-1149.989742	-1150.390786
**INT1_c6**	-1150.3303	0.393905	-1149.908	0.084474	-1149.987099	-1150.390368
**INT1_c7**	-1150.3326	0.393989	-1149.9103	0.085056	-1149.989449	-1150.390299
**INT1_c8**	-1150.3326	0.394017	-1149.9102	0.084957	-1149.989389	-1150.390294
**INT1_c9**	-1150.332	0.393853	-1149.9096	0.08537	-1149.989052	-1150.389639
**INT1_c10**	-1150.3325	0.393941	-1149.9102	0.085057	-1149.989462	-1150.38969
**INT1_c11**	-1150.3313	0.394082	-1149.9089	0.08612	-1149.98847	-1150.389265
**INT1_c12**	-1150.3308	0.393522	-1149.9087	0.085514	-1149.988149	-1150.388415
**INT1_c13**	-1150.3308	0.393535	-1149.9087	0.085473	-1149.988123	-1150.388403
**INT1_c14**	-1150.3312	0.393774	-1149.9091	0.085017	-1149.98821	-1150.388628
**INT1_c15**	-1150.3313	0.394046	-1149.909	0.084214	-1149.987712	-1150.389094
**INT1_c16**	-1150.3307	0.393762	-1149.9085	0.084657	-1149.987658	-1150.387931
**INT1_c17**	-1150.3307	0.393762	-1149.9085	0.084654	-1149.987655	-1150.387931
**INT1_c18**	-1150.3312	0.394157	-1149.9089	0.08371	-1149.987359	-1150.38805
**INT1_c19**	-1150.3297	0.393723	-1149.9077	0.08444	-1149.9866	-1150.386313
**INT1_c20**	-1150.3297	0.393716	-1149.9077	0.084466	-1149.986619	-1150.38627
**TS1**	-1150.2768	0.393127	-1149.8565	0.082945	-1149.933101	-1150.337611
**TS1_c2**	-1150.2767	0.393335	-1149.8564	0.081403	-1149.93218	-1150.337906
**TS1_c3**	-1150.2761	0.393071	-1149.8559	0.083192	-1149.932573	-1150.33664
**TS1_c4**	-1150.2761	0.393071	-1149.8559	0.083195	-1149.932574	-1150.336638
**TS1_c5**	-1150.2776	0.393484	-1149.8573	0.082244	-1149.933247	-1150.33726
**TS1_c6**	-1150.2777	0.393564	-1149.8573	0.08075	-1149.932754	-1150.337811
**TS1_c7**	-1150.2777	0.393566	-1149.8573	0.080745	-1149.932751	-1150.337811
**TS1_c8**	-1150.2783	0.393681	-1149.8578	0.080929	-1149.933344	-1150.337851
**TS1_c9**	-1150.279	0.393891	-1149.8585	0.080041	-1149.933523	-1150.33816
**TS1_c10**	-1150.2783	0.393635	-1149.8579	0.081453	-1149.933457	-1150.337438
**TS1_c11**	-1150.2783	0.393806	-1149.8578	0.080368	-1149.933072	-1150.337876
**TS1_c12**	-1150.2744	0.393182	-1149.8541	0.084095	-1149.931212	-1150.3357
**TS1_c13**	-1150.2761	0.393256	-1149.8558	0.082631	-1149.932213	-1150.336177

**TS1_c14**	-1150.2758	0.393414	-1149.8555	0.08117	-1149.931107	-1150.336921
**TS1_c15**	-1150.2744	0.393397	-1149.8539	0.083686	-1149.930864	-1150.335368
**TS1_c16**	-1150.2761	0.393631	-1149.8556	0.081001	-1149.931111	-1150.33649
**TS1_c17**	-1150.2761	0.393629	-1149.8556	0.080999	-1149.931113	-1150.336484
**TS1'**	-1150.277	0.393093	-1149.8571	0.079533	-1149.932318	-1150.336786
**TS1'_c2**	-1150.2754	0.392961	-1149.8555	0.080489	-1149.931257	-1150.335812
**TS1'_c3**	-1150.2772	0.393357	-1149.8572	0.079086	-1149.932038	-1150.336586
**TS1'_c4**	-1150.2749	0.393355	-1149.8547	0.08056	-1149.93031	-1150.335835
**TS1'_c5**	-1150.275	0.393299	-1149.8548	0.081398	-1149.930662	-1150.335451
**TS1'_c6**	-1150.2744	0.392991	-1149.8546	0.080525	-1149.930179	-1150.33511
**TS1'_c7**	-1150.2751	0.3936	-1149.8547	0.080648	-1149.930102	-1150.335563
**TS1'_c8**	-1150.2751	0.393601	-1149.8547	0.080644	-1149.9301	-1150.335563
**TS1'_c9**	-1150.2769	0.393342	-1149.8569	0.079431	-1149.931906	-1150.335613
**TS1'_c10**	-1150.2761	0.393228	-1149.8562	0.078953	-1149.931066	-1150.335464
**TS1'_c11**	-1150.2757	0.393349	-1149.8556	0.080141	-1149.930866	-1150.33472
**TS1'_c12**	-1150.273	0.393368	-1149.8526	0.081397	-1149.928562	-1150.334246
**TS1'_c13**	-1150.2724	0.393385	-1149.852	0.081401	-1149.927849	-1150.333425
**TS1'_c14**	-1150.2739	0.393243	-1149.8539	0.079933	-1149.929139	-1150.333297
**TS1'_c15**	-1150.2729	0.393603	-1149.8524	0.080777	-1149.927904	-1150.333067
**TS1'_c16**	-1150.2724	0.393257	-1149.852	0.08173	-1149.928237	-1150.332206
**TS1'_c17**	-1150.2724	0.39332	-1149.8521	0.081529	-1149.92799	-1150.332505
**TS1'_c18**	-1150.2691	0.393411	-1149.8487	0.081722	-1149.924656	-1150.331706
**TS1'_c19**	-1150.2728	0.393208	-1149.8529	0.07951	-1149.92778	-1150.331998
**TS1'_c20**	-1150.3207	0.395907	-1149.8977	0.082294	-1149.974118	-1150.377395
**INT2**	-1150.3319	0.395984	-1149.9085	0.085376	-1149.986308	-1150.393933
**INT2_c2**	-1150.3319	0.396	-1149.9085	0.084812	-1149.986025	-1150.393929
**INT2_c3**	-1150.3338	0.39587	-1149.9105	0.083813	-1149.987578	-1150.393673
**INT2_c4**	-1150.333	0.395869	-1149.9098	0.084031	-1149.986989	-1150.393208
**INT2_c5**	-1150.3319	0.395965	-1149.9094	0.08097	-1149.984512	-1150.393937
**INT2_c6**	-1150.3348	0.396446	-1149.9113	0.081752	-1149.987238	-1150.393877
**INT2_c7**	-1150.3331	0.396494	-1149.9095	0.081989	-1149.98572	-1150.393681
**INT2_c8**	-1150.335	0.396326	-1149.9115	0.082536	-1149.988012	-1150.393246
**INT2_c9**	-1150.3334	0.396357	-1149.9098	0.083075	-1149.98643	-1150.393096

**INT2_c10**	-1150.3334	0.396357	-1149.9098	0.083089	-1149.986436	-1150.393068
**INT2_c11**	-1150.3336	0.396406	-1149.9099	0.083338	-1149.986666	-1150.392856
**INT2_c12**	-1150.3349	0.396601	-1149.9113	0.081487	-1149.987218	-1150.39365
**INT2_c13**	-1150.3345	0.396587	-1149.9108	0.081552	-1149.986863	-1150.393266
**INT2_c14**	-1150.3345	0.39639	-1149.9109	0.082667	-1149.987503	-1150.392511
**INT2_c15**	-1150.3333	0.396426	-1149.9097	0.082443	-1149.986177	-1150.391986
**INT2_c16**	-1150.3358	0.396598	-1149.9123	0.080763	-1149.987834	-1150.392347
**INT2_c17**	-1150.3328	0.396595	-1149.9091	0.081986	-1149.985202	-1150.391772
**INT2_c18**	-1150.3328	0.396595	-1149.9091	0.081983	-1149.985201	-1150.39177
**INT2_c19**	-1150.3328	0.396595	-1149.9091	0.081982	-1149.9852	-1150.39177
**INT2_c20**	-1150.333	0.395858	-1149.9098	0.082735	-1149.986457	-1150.390447
**INT2'**	-1150.3271	0.395611	-1149.9042	0.083694	-1149.981292	-1150.390396
**INT2'_c2**	-1150.3287	0.396032	-1149.9055	0.082125	-1149.981971	-1150.39121
**INT2'_c3**	-1150.3289	0.396058	-1149.9058	0.082313	-1149.982176	-1150.391002
**INT2'_c4**	-1150.3296	0.396291	-1149.9063	0.081437	-1149.982312	-1150.391251
**INT2'_c5**	-1150.3301	0.396154	-1149.9069	0.081941	-1149.983105	-1150.390921
**INT2'_c6**	-1150.3305	0.39628	-1149.9073	0.080999	-1149.983015	-1150.391068
**INT2'_c7**	-1150.3305	0.396479	-1149.9071	0.080694	-1149.982686	-1150.391351
**INT2'_c8**	-1150.3305	0.39648	-1149.9071	0.080689	-1149.982683	-1150.391352
**INT2'_c9**	-1150.3274	0.396	-1149.9042	0.082739	-1149.981009	-1150.389648
**INT2'_c10**	-1150.3278	0.395752	-1149.9048	0.082054	-1149.981215	-1150.389353
**INT2'_c11**	-1150.3272	0.395872	-1149.9039	0.083814	-1149.981299	-1150.388538
**INT2'_c12**	-1150.3273	0.395857	-1149.904	0.083357	-1149.981233	-1150.388441
**INT2'_c13**	-1150.3286	0.395676	-1149.9056	0.083072	-1149.982552	-1150.38812
**INT2'_c14**	-1150.3276	0.395928	-1149.9045	0.082873	-1149.981344	-1150.388122
**INT2'_c15**	-1150.328	0.396002	-1149.9049	0.081938	-1149.981238	-1150.388399
**INT2'_c16**	-1150.328	0.396003	-1149.9049	0.081939	-1149.981237	-1150.388398
**INT2'_c17**	-1150.328	0.396004	-1149.9049	0.081932	-1149.981233	-1150.3884
**INT2'_c18**	-1150.328	0.396003	-1149.9049	0.08194	-1149.981237	-1150.388396
**INT2'_c19**	-1150.3268	0.39593	-1149.9036	0.082212	-1149.980077	-1150.386844
**INT2'_c20**	-1150.3289	0.396485	-1149.9055	0.079987	-1149.980976	-1150.387847
**TS2a**	-1150.2788	0.393432	-1149.8583	0.082563	-1149.934589	-1150.340712
**TS2a_c2**	-1150.2785	0.393549	-1149.8579	0.082262	-1149.934068	-1150.340112

**TS2a_c3**	-1150.2787	0.393555	-1149.8581	0.082042	-1149.9342	-1150.340019
**TS2a_c4**	-1150.2799	0.39361	-1149.8594	0.080954	-1149.935026	-1150.340329
**TS2a_c5**	-1150.2782	0.393583	-1149.8576	0.081918	-1149.933536	-1150.339832
**TS2a_c6**	-1150.278	0.393341	-1149.8575	0.082624	-1149.933893	-1150.339256
**TS2a_c7**	-1150.2782	0.393281	-1149.8577	0.082853	-1149.934348	-1150.338913
**TS2a_c8**	-1150.279	0.393658	-1149.8585	0.081687	-1149.934253	-1150.339818
**TS2a_c9**	-1150.2781	0.393354	-1149.8576	0.082803	-1149.934158	-1150.338916
**TS2a_c10**	-1150.2776	0.393581	-1149.857	0.082512	-1149.933257	-1150.338904
**TS2a_c11**	-1150.2792	0.393658	-1149.8587	0.0808	-1149.934187	-1150.339335
**TS2a_c12**	-1150.2774	0.39342	-1149.8569	0.08244	-1149.933099	-1150.338533
**TS2a_c13**	-1150.2783	0.393622	-1149.8577	0.081796	-1149.933566	-1150.338647
**TS2a_c14**	-1150.2769	0.393056	-1149.8565	0.083148	-1149.933308	-1150.337498
**TS2a_c15**	-1150.2799	0.393894	-1149.8593	0.079818	-1149.934233	-1150.339429
**TS2a_c16**	-1150.2777	0.393412	-1149.8574	0.081083	-1149.933089	-1150.33786
**TS2a_c17**	-1150.2789	0.393663	-1149.8583	0.081598	-1149.934216	-1150.337755
**TS2a_c18**	-1150.2768	0.393552	-1149.8563	0.081306	-1149.931963	-1150.33771
**TS2a_c19**	-1150.2801	0.393885	-1149.8596	0.07981	-1149.934442	-1150.337841
**TS2a_c20**	-1150.2784	0.393621	-1149.858	0.080804	-1149.933343	-1150.33639
**TS2b**	-1150.2797	0.393385	-1149.8596	0.080076	-1149.934771	-1150.338496
**TS2b_c2**	-1150.2778	0.393669	-1149.8573	0.082125	-1149.933183	-1150.337204
**TS2b_c3**	-1150.2778	0.39367	-1149.8573	0.08212	-1149.93318	-1150.337204
**TS2b_c4**	-1150.2778	0.393673	-1149.8573	0.082109	-1149.933174	-1150.337204
**TS2b_c5**	-1150.2778	0.393672	-1149.8573	0.082109	-1149.933174	-1150.337204
**TS2b_c6**	-1150.2796	0.393416	-1149.8595	0.079716	-1149.934381	-1150.337641
**TS2b_c7**	-1150.2771	0.393519	-1149.8567	0.082105	-1149.932655	-1150.336601
**TS2b_c8**	-1150.2764	0.393477	-1149.8559	0.082627	-1149.932144	-1150.336289
**TS2b_c9**	-1150.2764	0.393476	-1149.8559	0.082627	-1149.932144	-1150.336289
**TS2b_c10**	-1150.2775	0.393558	-1149.8571	0.08211	-1149.933075	-1150.336407
**TS2b_c11**	-1150.2775	0.393558	-1149.8571	0.082106	-1149.933073	-1150.336407
**TS2b_c12**	-1150.2775	0.393558	-1149.8571	0.082105	-1149.933072	-1150.336407
**TS2b_c13**	-1150.2781	0.393354	-1149.858	0.079885	-1149.933013	-1150.336448
**TS2b_c14**	-1150.2779	0.393868	-1149.8574	0.079911	-1149.932328	-1150.336871
**TS2b_c15**	-1150.2759	0.393686	-1149.8554	0.082007	-1149.931316	-1150.335155

**TS2b_c16**	-1150.2779	0.39367	-1149.8576	0.080179	-1149.932678	-1150.335317
**TS2b_c17**	-1150.2771	0.394053	-1149.8563	0.080183	-1149.931395	-1150.335645
**TS2b_c18**	-1150.2756	0.393729	-1149.8549	0.081246	-1149.930588	-1150.334871
**TS2b_c19**	-1150.278	0.393338	-1149.8581	0.078637	-1149.932622	-1150.334514
**TS2b_c20**	-1150.278	0.393338	-1149.8581	0.078633	-1149.93262	-1150.334514
**TS2a'**	-1150.2739	0.39301	-1149.8539	0.081813	-1149.930022	-1150.337612
**TS2a'_c2**	-1150.2739	0.393011	-1149.8539	0.081811	-1149.93002	-1150.337608
**TS2a'_c3**	-1150.2739	0.393011	-1149.8539	0.081812	-1149.93002	-1150.337608
**TS2a'_c4**	-1150.2739	0.393012	-1149.8539	0.081803	-1149.930017	-1150.337606
**TS2a'_c5**	-1150.2733	0.393202	-1149.853	0.081616	-1149.929027	-1150.337503
**TS2a'_c6**	-1150.2747	0.393387	-1149.8545	0.08141	-1149.930266	-1150.337441
**TS2a'_c7**	-1150.2744	0.393388	-1149.8542	0.081681	-1149.930064	-1150.337326
**TS2a'_c8**	-1150.2746	0.393248	-1149.8544	0.081334	-1149.930311	-1150.337067
**TS2a'_c9**	-1150.2759	0.393572	-1149.8556	0.080702	-1149.930978	-1150.33753
**TS2a'_c10**	-1150.2745	0.393291	-1149.8543	0.080662	-1149.929966	-1150.337017
**TS2a'_c11**	-1150.2744	0.393626	-1149.854	0.080131	-1149.929231	-1150.337584
**TS2a'_c12**	-1150.2744	0.393624	-1149.854	0.080135	-1149.929234	-1150.337567
**TS2a'_c13**	-1150.2738	0.393313	-1149.8536	0.080672	-1149.929193	-1150.336951
**TS2a'_c14**	-1150.2727	0.39358	-1149.8522	0.080682	-1149.927753	-1150.336696
**TS2a'_c15**	-1150.2729	0.393488	-1149.8526	0.080283	-1149.927997	-1150.336134
**TS2a'_c16**	-1150.2717	0.39312	-1149.8515	0.083073	-1149.928088	-1150.33458
**TS2a'_c17**	-1150.27	0.393178	-1149.8498	0.081995	-1149.925875	-1150.334843
**TS2a'_c18**	-1150.2712	0.39305	-1149.8511	0.081544	-1149.926964	-1150.332731
**TS2a'_c19**	-1150.2712	0.39305	-1149.8511	0.081541	-1149.926964	-1150.33273
**TS2a'_c20**	-1150.2712	0.393053	-1149.8511	0.08153	-1149.926957	-1150.33273
**TS2b'**	-1150.2727	0.393111	-1149.8525	0.083418	-1149.929295	-1150.333969
**TS2b'_c2**	-1150.2744	0.393808	-1149.8539	0.080469	-1149.929155	-1150.335147
**TS2b'_c3**	-1150.2747	0.393269	-1149.8548	0.079316	-1149.929683	-1150.334933
**TS2b'_c4**	-1150.2714	0.393253	-1149.8511	0.082865	-1149.92754	-1150.333661
**TS2b'_c5**	-1150.2737	0.393426	-1149.8534	0.081331	-1149.929165	-1150.334208
**TS2b'_c6**	-1150.2726	0.393175	-1149.8524	0.082435	-1149.928732	-1150.333424
**TS2b'_c7**	-1150.2742	0.393177	-1149.8542	0.080107	-1149.929512	-1150.333229
**TS2b'_c8**	-1150.2688	0.392963	-1149.8488	0.08371	-1149.925739	-1150.331315

**TS2b'_c9**	-1150.2691	0.393385	-1149.8487	0.082464	-1149.924906	-1150.33131
**TS2b'_c10**	-1150.2705	0.393598	-1149.85	0.080692	-1149.925592	-1150.331755
**TS2b'_c11**	-1150.2679	0.393392	-1149.8475	0.082383	-1149.923669	-1150.330738
**TS2b'_c12**	-1150.2729	0.394042	-1149.8523	0.078559	-1149.926773	-1150.332517
**TS2b'_c13**	-1150.2726	0.393916	-1149.852	0.079383	-1149.926966	-1150.332076
**TS2b'_c14**	-1150.2693	0.393743	-1149.8488	0.079856	-1149.923926	-1150.330997
**TS2b'_c15**	-1150.2693	0.393746	-1149.8488	0.079848	-1149.92392	-1150.330996
**TS2b'_c16**	-1150.2693	0.393747	-1149.8488	0.079846	-1149.923919	-1150.330997
**TS2b'_c17**	-1150.2693	0.393748	-1149.8488	0.079842	-1149.923917	-1150.330998
**TS2b'_c18**	-1150.2693	0.393749	-1149.8488	0.079837	-1149.923914	-1150.330998
**TS2b'_c19**	-1150.2693	0.39375	-1149.8488	0.079835	-1149.923912	-1150.330999
**TS2b'_c20**	-1150.2689	0.393474	-1149.8485	0.081093	-1149.924217	-1150.330188
**INT3**	-1150.3285	0.392765	-1149.9068	0.087888	-1149.987675	-1150.392516
**INT3_c2**	-1150.3322	0.393079	-1149.9103	0.087441	-1149.990814	-1150.391628
**INT3_c3**	-1150.3324	0.393213	-1149.9104	0.086925	-1149.990614	-1150.391823
**INT3_c4**	-1150.3329	0.393282	-1149.911	0.086246	-1149.990871	-1150.391921
**INT3_c5**	-1150.3328	0.393326	-1149.9108	0.086275	-1149.990765	-1150.391825
**INT3_c6**	-1150.3328	0.393324	-1149.9108	0.086281	-1149.990769	-1150.39182
**INT3_c7**	-1150.3332	0.393507	-1149.9112	0.086033	-1149.990821	-1150.391564
**INT3_c8**	-1150.3326	0.39354	-1149.9105	0.086305	-1149.990283	-1150.391269
**INT3_c9**	-1150.3341	0.3937	-1149.912	0.08457	-1149.990987	-1150.391912
**INT3_c10**	-1150.3312	0.393854	-1149.9089	0.086301	-1149.988598	-1150.391094
**INT3_c11**	-1150.3319	0.393173	-1149.9101	0.086137	-1149.989875	-1150.39029
**INT3_c12**	-1150.3328	0.393591	-1149.9107	0.085957	-1149.99039	-1150.390638
**INT3_c13**	-1150.3298	0.393341	-1149.9078	0.086405	-1149.987643	-1150.389966
**INT3_c14**	-1150.3306	0.393572	-1149.9085	0.085029	-1149.987807	-1150.390488
**INT3_c15**	-1150.3306	0.393574	-1149.9085	0.085023	-1149.987803	-1150.39049
**INT3_c16**	-1150.3317	0.393668	-1149.9096	0.084546	-1149.988665	-1150.390311
**INT3_c17**	-1150.3314	0.39393	-1149.9091	0.084491	-1149.988026	-1150.389428
**INT3_c18**	-1150.3307	0.393924	-1149.9083	0.084787	-1149.987484	-1150.389272
**INT3_c19**	-1150.3307	0.393926	-1149.9083	0.084782	-1149.98748	-1150.389272
**INT3_c20**	-1150.3314	0.393942	-1149.9091	0.084415	-1149.987988	-1150.389433
**PRD_c1**	-575.15267	0.195984	-574.94254	0.052692	-574.992475	-575.1917012

**PRD_c2**	-575.15267	0.195985	-574.94254	0.052685	-574.992472	-575.1916942
**PRD_c3**	-575.15224	0.196085	-574.94199	0.053313	-574.992283	-575.1908133
**PRD_c4**	-575.15224	0.196086	-574.94199	0.053296	-574.992274	-575.1908092
**PRD_c5**	-575.15089	0.195783	-574.94073	0.054104	-574.991648	-575.1900387
**PRD_c6**	-575.15143	0.195941	-574.94138	0.053059	-574.991502	-575.1904693
**PRD_c7**	-575.15143	0.195941	-574.94138	0.053053	-574.991499	-575.190469
**PRD_c8**	-575.15143	0.195941	-574.94138	0.053052	-574.991498	-575.1904689
**PRD_c9**	-575.15143	0.195941	-574.94138	0.053057	-574.9915	-575.1904663
**PRD_c10**	-575.15143	0.195942	-574.94138	0.053056	-574.9915	-575.1904659
**PRD_c11**	-575.15143	0.195941	-574.94138	0.053051	-574.991497	-575.1904677
**PRD_c12**	-575.15198	0.195807	-574.94201	0.053006	-574.992197	-575.1894769
**PRD_c13**	-575.15198	0.195808	-574.94201	0.053004	-574.992196	-575.189476
**PRD_c14**	-575.15375	0.196301	-574.94353	0.051012	-574.992708	-575.1897872
**PRD_c15**	-575.15211	0.195787	-574.94207	0.053295	-574.992608	-575.1882257
**PRD_c16**	-575.15211	0.195788	-574.94207	0.053293	-574.992607	-575.1882256
**PRD_c17**	-575.15302	0.196311	-574.94277	0.051858	-574.992142	-575.1895972
**PRD_c18**	-575.15302	0.196313	-574.94277	0.05185	-574.992137	-575.1895973
**PRD_c19**	-575.15278	0.196062	-574.94283	0.05074	-574.991878	-575.1892959
**PRD_c20**	-575.15278	0.196062	-574.94283	0.05074	-574.991878	-575.1892948

Benchmarking – SMD($CHCl_3$)-M06-2X/def2-TZVP//M06-2X/def2-SVP

Structure	E/au	ZPE/au	H/au	T.S/au	qh-G/au	SP
**INT1'-4m**	-1150.329	0.393618	-1149.9067	0.08598	-1149.986455	-1151.662602
**TS1'-4m**	-1150.2454	0.391028	-1149.8261	0.085033	-1149.905151	-1151.585289
**INT2'-4m**	-1150.3162	0.394549	-1149.8938	0.08464	-1149.972012	-1151.648587
**INT1-4m**	-1150.3243	0.393211	-1149.9021	0.088177	-1149.98295	-1151.657663
**TS1-4m**	-1150.2471	0.390691	-1149.8281	0.084053	-1149.906693	-1151.584067
**TS2a-4m**	-1150.2477	0.39169	-1149.8279	0.08523	-1149.906621	-1151.582574
**INT3a-4m**	-1150.3233	0.393239	-1149.9019	0.085431	-1149.980956	-1151.657972
**TS2b-4m**	-1150.2469	0.3922	-1149.827	0.081639	-1149.903938	-1151.579805
**INT3b-4m**	-1150.3126	0.393996	-1149.8899	0.085819	-1149.969792	-1151.648353
**INT1-4m**	-1150.3243	0.393211	-1149.9021	0.088177	-1149.98295	-1151.657663

Benchmarking – SMD($CHCl_3$)-DLPNO-CCSD(T)/CBS//M06-2X/def2-SVP

Structure	E/au	ZPE/au	H/au	T.S/au	qh-G/au	SP
**INT1'-4m**	-1150.329	0.393618	-1149.9067	0.08598	-1149.986455	-1150.390716
**TS1'-4m**	-1150.2454	0.391028	-1149.8261	0.085033	-1149.905151	-1150.312855
**INT2'-4m**	-1150.3162	0.394549	-1149.8938	0.08464	-1149.972012	-1150.376928
**TS2b'-4m**	-1150.249	0.391513	-1149.8295	0.084244	-1149.907763	-1150.315139
**INT3b'-4m**	-1150.3264	0.392884	-1149.9044	0.088759	-1149.98555	-1150.389108
**TS2a'-4m**	-1150.2333	0.390099	-1149.8146	0.087429	-1149.894822	-1150.29994
**INT3a'-4m**	-1150.3243	0.392503	-1149.9026	0.088233	-1149.984042	-1150.393586
**INT1-4m**	-1150.3243	0.393211	-1149.9021	0.088177	-1149.98295	-1150.387481
**TS1-4m**	-1150.2471	0.390691	-1149.8281	0.084053	-1149.906693	-1150.310898
**INT2-4m**	-1150.323	0.395339	-1149.8999	0.085211	-1149.977927	-1150.382758
**TS2a-4m**	-1150.2477	0.39169	-1149.8279	0.08523	-1149.906621	-1150.312956
**INT3a-4m**	-1150.3233	0.393239	-1149.9019	0.085431	-1149.980956	-1150.387781
**TS2b-4m**	-1150.2469	0.3922	-1149.827	0.081639	-1149.903938	-1150.307512
**INT3b-4m**	-1150.3126	0.393996	-1149.8899	0.085819	-1149.969792	-1150.377888
**INT1'-4m**	-1150.329	0.393618	-1149.9067	0.08598	-1149.986455	-1150.390716
**TS1'-4m**	-1150.2454	0.391028	-1149.8261	0.085033	-1149.905151	-1150.312855
**INT2'-4m**	-1150.3162	0.394549	-1149.8938	0.08464	-1149.972012	-1150.376928
**TS2b'-4m**	-1150.249	0.391513	-1149.8295	0.084244	-1149.907763	-1150.315139
**INT3b'-4m**	-1150.3264	0.392884	-1149.9044	0.088759	-1149.98555	-1150.389108
**TS2a'-4m**	-1150.2333	0.390099	-1149.8146	0.087429	-1149.894822	-1150.29994
**INT3a'-4m**	-1150.3243	0.392503	-1149.9026	0.088233	-1149.984042	-1150.393586
**INT1-4m**	-1150.3243	0.393211	-1149.9021	0.088177	-1149.98295	-1150.387481
**TS1-4m**	-1150.2471	0.390691	-1149.8281	0.084053	-1149.906693	-1150.310898
**INT2-4m**	-1150.323	0.395339	-1149.8999	0.085211	-1149.977927	-1150.382758
**TS2a-4m**	-1150.2477	0.39169	-1149.8279	0.08523	-1149.906621	-1150.312956
**INT3a-4m**	-1150.3233	0.393239	-1149.9019	0.085431	-1149.980956	-1150.387781
**TS2b-4m**	-1150.2469	0.3922	-1149.827	0.081639	-1149.903938	-1150.307512
**INT3b-4m**	-1150.3126	0.393996	-1149.8899	0.085819	-1149.969792	-1150.377888

Benchmarking – SMD($CHCl_3$)-M06-2X/def2-TZVP//SMD($CHCl_3$)-M06-2X/def2-SVP

Structure	E/au	ZPE/au	H/au	T.S/au	qh-G/au	SP
**MAA_solv**	-535.90554	0.166733	-535.72589	0.049209	-535.77298	-536.5217086
**PNA_solv**	-614.42445	0.223517	-614.18512	0.057751	-614.2393	-615.131465
**INT1-4m_solv**	-1150.3435	0.392231	-1149.9223	0.088313	-1150.0032	-1151.658284
**TS1-4m_solv**	-1150.2666	0.38961	-1149.8485	0.084517	-1149.9276	-1151.585253
**INT2-4m_solv**	-1150.3445	0.394517	-1149.922	0.087035	-1150.0011	-1151.658282
**TS2b-4m_solv**	-1150.265	0.390786	-1149.8463	0.083146	-1149.9241	-1151.580547
**INT3b-4m_solv**	-1150.3328	0.393326	-1149.9109	0.085545	-1149.9905	-1151.648936
**TS2a-4m_solv**	-1150.2673	0.390401	-1149.8486	0.086109	-1149.9279	-1151.585416
**INT3a-4m_solv**	-1150.3432	0.392117	-1149.9218	0.089632	-1150.0035	-1151.658568
**PRD_solv**	-575.16337	0.19541	-574.95362	0.053838	-575.0044	-575.82498

Benchmarking – SMD($CHCl_3$)-M06-2X/6-311++G(d,p)//M06-2X/6-31+G(d)

Structure	E/au	ZPE/au	H/au	T.S/au	qh-G/au	SP
**MAA**	-536.30824	0.167878	-536.12743	0.049372	-536.17456	-536.463194
**PNA**	-614.88502	0.225211	-614.64401	0.057987	-614.69825	-615.067343
**INT1'_4m**	-1151.2142	0.395513	-1150.7899	0.08655	-1150.87	-1151.54464
**TS1'_4m**	-1151.133	0.392953	-1150.7117	0.085756	-1150.7911	-1151.468593
**INT2'_4m**	-1151.2004	0.396465	-1150.776	0.085004	-1150.8544	-1151.529199
**INT1_4m**	-1151.2081	0.394938	-1150.7841	0.088664	-1150.8652	-1151.539029
**TS1_4m**	-1151.1334	0.392514	-1150.7124	0.085471	-1150.7918	-1151.466316
**INT2_4m**	-1151.2103	0.397318	-1150.785	0.087053	-1150.8641	-1151.537704
**TS2a_4m**	-1151.1347	0.393436	-1150.713	0.085758	-1150.7921	-1151.466351
**INT3a_4m**	-1151.2085	0.39477	-1150.7845	0.089231	-1150.866	-1151.540108
**TS2b_4m**	-1151.1314	0.393784	-1150.7098	0.082651	-1150.7873	-1151.462661
**INT3b_4m**	-1151.1975	0.395548	-1150.7731	0.087056	-1150.8537	-1151.530317

Acid catalysis

Structure	E/au	ZPE/au	H/au	T.S/au	qh-G/au	SP
**TS_RCT**	-1150.6876	0.403987	-1150.2542	0.092402	-1150.337338	-1150.796388

**TS_acid_prime**	-1150.6261	0.402294	-1150.1941	0.090672	-1150.276776	-1150.750628
**TS_INT**	-1150.6545	0.403093	-1150.2207	0.094493	-1150.306029	-1150.773914
**TS_acid**	-1150.6364	0.402793	-1150.2041	0.089929	-1150.286391	-1150.757693
**TS_acid_c2**	-1150.6338	0.402885	-1150.2014	0.08919	-1150.283329	-1150.756322
**TS_acid_c3**	-1150.6358	0.40309	-1150.2035	0.087809	-1150.284576	-1150.755819
**TS_acid_c4**	-1150.6348	0.403154	-1150.2024	0.087654	-1150.28354	-1150.755425
**TS_acid_c5**	-1150.6308	0.402955	-1150.1985	0.087669	-1150.279845	-1150.754766
**TS_acid_c6**	-1150.6346	0.403361	-1150.2022	0.085342	-1150.282452	-1150.755551
**TS_acid_c7**	-1150.6375	0.40358	-1150.2049	0.08616	-1150.285226	-1150.75555
**TS_acid_c8**	-1150.6351	0.403904	-1150.2023	0.085431	-1150.282287	-1150.755016
**TS_acid_c9**	-1150.6351	0.403903	-1150.2023	0.085432	-1150.282287	-1150.755013
**TS_acid_c10**	-1150.6362	0.403528	-1150.2036	0.086719	-1150.284173	-1150.753653
**TS_acid_c11**	-1150.6356	0.403072	-1150.2036	0.085476	-1150.28367	-1150.753441
**TS_acid_c12**	-1150.6332	0.403657	-1150.2005	0.085636	-1150.280716	-1150.752696

IV. Supplementary References:

Full reference Gaussian 16:

Gaussian 16, Revision B.01, Frisch, M. J.; Trucks, G. W.; Schlegel, H. B.; Scuseria, G. E.; Robb, M. A.; Cheeseman, J. R.; Scalmani, G.; Barone, V.; Mennucci, B.; Petersson, G. A.; Nakatsuji, H.; Caricato, M.; Li, X.; Hratchian, H. P.; Izmaylov, A. F.; Bloino, J.; Zheng, G.; Sonnenberg, J. L.; Hada, M.; Ehara, M.; Toyota, K.; Fukuda, R.; Hasegawa, J.; Ishida, M.; Nakajima, T.; Honda, Y.; Kitao, O.; Nakai, H.; Vreven, T.; Montgomery Jr., J. A.; Peralta, J. E.; Ogliaro, F.; Bearpark, M.; Heyd, J. J.; Brothers, E.; Kudin, K. N.; Staroverov, V. N.; Kobayashi, R.; Normand, J.; Raghavachari, K.; Rendell, A.; Burant, J. C.; Iyengar, S. S.; Tomasi, J.; Cossi, M.; Rega, N.; Millam, J. M.; Klene, M.; Knox, J. E.; Cross, J. B.; Bakken, V.; Adamo, C.; Jaramillo, J.; Gomperts, R.; Stratmann, R. E.; Yazyev, O.; Austin, A. J.; Cammi, R.; Pomelli, C.; Ochterski, J. W.; Martin, R. L.; Morokuma, K.; Zakrzewski, V. G.; Voth, G. A.; Salvador, P.; Dannenberg, J. J.; Dapprich, S.; Daniels, A. D.; Farkas, Ö.; Foresman, J. B.; Ortiz, J. V; Cioslowski, J.; Fox, D. J. Gaussian, Inc., Wallingford CT, 2016.

(1) Zhao, Y.; Truhlar, D. G. The M06 Suite of Density Functionals for Main Group Thermochemistry, Thermochemical Kinetics, Noncovalent Interactions, Excited States, and Transition Elements: Two New Functionals and Systematic Testing of Four M06-Class Functionals and 12 Other Function. *Theor. Chem. Acc.* **2008**, *120* (1), 215–241.

(2) Weigend, F.; Ahlrichs, R. Balanced Basis Sets of Split Valence, Triple Zeta Valence and Quadruple Zeta Valence Quality for H to Rn: Design and Assessment of Accuracy. *Phys. Chem. Chem. Phys.* **2005**, *7* (18), 3297–3305.

(3) Weigend, F. Accurate Coulomb-Fitting Basis Sets for H to Rn. *Phys. Chem. Chem. Phys.* **2006**, *8* (9), 1057–1065.

(4) Ditchfield, R.; Hehre, W. J.; Pople, J. A. Self-Consistent Molecular-Orbital Methods. IX. An Extended Gaussian-Type Basis for Molecular-Orbital Studies of Organic Molecules. *J. Chem. Phys.* **2004**, *54* (2), 724–728.

(5) Marenich, A. V.; Cramer, C. J.; Truhlar, D. G. Universal Solvation Model Based on Solute Electron Density and on a Continuum Model of the Solvent Defined by the Bulk Dielectric Constant and Atomic Surface Tensions. *J. Phys. Chem. B* **2009**, *113* (18), 6378–6396.

(6) Frisch, M. J. .; Trucks, G. W. .; Schlegel, H. B. .; Scuseria, G. E. .; Robb, M. A. .; Cheeseman, J. R. .; Scalmani, G. .; Barone, V. .; Petersson, G. A. .; Nakatsuji, H. .; et al. Gaussian 16, Revision B.01. 2016.

(7) Riplinger, C.; Neese, F. An Efficient and near Linear Scaling Pair Natural Orbital Based Local Coupled Cluster Method. *J. Chem. Phys.* **2013**, *138* (3), 034106.

(8) Riplinger, C.; Sandhoefer, B.; Hansen, A.; Neese, F. Natural Triple Excitations in Local Coupled Cluster Calculations with Pair Natural Orbitals. *J. Chem. Phys.* **2013**, *139* (13), 134101.

(9) Neese, F. The ORCA Program System. *Wiley Interdiscip. Rev. Comput. Mol. Sci.* **2012**, *2* (1), 73–78.

(10) Neese, F. Software Update: The ORCA Program System, Version 4.0. *Wiley Interdiscip. Rev. Comput. Mol. Sci.* **2018**, *8* (1), e1327.

(11) Neese, F.; Wennmohs, F.; Becker, U.; Riplinger, C. The ORCA Quantum Chemistry Program Package. *J. Chem. Phys.* **2020**, *152* (22), 224108.

(12) Guo, Y.; Riplinger, C.; Becker, U.; Liakos, D. G.; Minenkov, Y.; Cavallo, L.; Neese, F. An Improved Linear Scaling Perturbative Triples Correction for the Domain Based Local Pair-Natural Orbital Based Singles and Doubles Coupled Cluster Method [DLPNO-CCSD(T)]. *J. Chem. Phys.* **2018**, *148* (1), 011101.

(13) Kollmar, C. The Role of Energy Denominators in Self-consistent Field (SCF) Calculations for Open Shell Systems. *J. Chem. Phys.* **1996**, *105* (18), 8204–8212.

(14) Halkier, A.; Helgaker, T.; Jørgensen, P.; Klopper, W.; Koch, H.; Olsen, J.; Wilson, A. K. Basis-Set Convergence in Correlated Calculations on Ne, N 2 , and H 2 O. *Chem. Phys. Lett.* **1998**, *286* (3–4), 243–252.

(15) Helgaker, T.; Klopper, W.; Koch, H.; Noga, J. Basis-Set Convergence of Correlated Calculations on Water. *J. Chem. Phys.* **1997**, *106* (23), 9639–9646.

(16) Halkier, A.; Helgaker, T.; Jørgensen, P.; Klopper, W.; Olsen, J. Basis-Set Convergence of the Energy in Molecular Hartree-Fock Calculations. *Chem. Phys. Lett.* **1999**, *302* (5–6), 437–446.

(17) Dunning, T. H. Gaussian Basis Sets for Use in Correlated Molecular Calculations. I. The Atoms Boron through Neon and Hydrogen. *J. Chem. Phys.* **1989**, *90* (2), 1007–1023.

(18) Woon, D. E.; Dunning, T. H. Gaussian Basis Sets for Use in Correlated Molecular

Calculations. III. The Atoms Aluminum through Argon. *J. Chem. Phys.* **1993**, *98* (2), 1358–1371.

(19) Woon, D. E.; Dunning, T. H. Gaussian Basis Sets for Use in Correlated Molecular Calculations. V. Core-valence Basis Sets for Boron through Neon. *J. Chem. Phys.* **1995**, *103* (11), 4572–4585.

(20) Stoychev, G. L.; Auer, A. A.; Neese, F. Automatic Generation of Auxiliary Basis Sets. *J. Chem. Theory Comput.* **2017**, *13* (2), 554–562.

(21) Schrödinger, L. *The PyMOL Molecular Graphics Development Component, Version 1.8*; 2015.

(22) Tillman, K. R.; Meacham, R.; Highmoore, J. F.; Barankovich, M.; Witkowski, A. M.; Mather, P. T.; Graf, T.; Shipp, D. A.; Rolsma, A. N.; Barankovich, M.; et al. Dynamic Covalent Exchange in Poly(Thioether Anhydrides). *Polym. Chem.* **2020**, *11* (47), 7551–7561.

(23) Needleman, S. B.; Wunsch, C. D. A General Method Applicable to the Search for Similarities in the Amino Acid Sequence of Two Proteins. *J. Mol. Biol.* **1970**, *48* (3), 443–453.

(24) Henikoff, S.; Henikoff, J. G. Amino Acid Substitution Matrices from Protein Blocks. *Proc. Natl. Acad. Sci. U. S. A.* **1992**, *89* (22), 10915–10919.

(25) Kabsch, W. A Solution for the Best Rotation to Relate Two Sets of Vectors. *Acta Cryst.* **1976**, *32* (5), 922–923.

(26) Liakos, D. G.; Sparta, M.; Kesharwani, M. K.; Martin, J. M. L.; Neese, F. Exploring the Accuracy Limits of Local Pair Natural Orbital Coupled-Cluster Theory. *J. Chem. Theory Comput.* **2015**, *11* (4), 1525–1539.

(27) Mallick, S.; Roy, B.; Kumar, P. A Comparison of DLPNO-CCSD(T) and CCSD(T) Method for the Determination of the Energetics of Hydrogen Atom Transfer Reactions. *Comput. Theor. Chem.* **2020**, *1187*, 112934.

(28) Clarke, R. W.; McGraw, M. L.; Newell, B. S.; Chen, E. Y.-X. Thermomechanical Activation Achieving Orthogonal Working/Healing Conditions of Nanostructured Tri-Block Copolymer Thermosets. *Cell Reports Phys. Sci.* **2021**, *2* (7), 100483.

(29) Pracht, P.; Bauer, C. A.; Grimme, S. Automated and Efficient Quantum Chemical Determination and Energetic Ranking of Molecular Protonation Sites. *J. Comput. Chem.* **2017**, *38* (30), 2618–2631.

(30) Ess, D. H.; Houk, K. N. Distortion/Interaction Energy Control of 1,3-Dipolar Cycloaddition Reactivity. *J. Am. Chem. Soc.* **2007**, *129* (35), 10646–10647.

(31) Fernández, I.; Bickelhaupt, F. M. The Activation Strain Model and Molecular Orbital Theory: Understanding and Designing Chemical Reactions. *Chem. Soc. Rev.* **2014**, *43* (14), 4953–4967.

(32) Bickelhaupt, F. M.; Houk, K. N. Analyzing Reaction Rates with the Distortion/Interaction-Activation Strain Model. *Angew. Chem. Int. Ed.* **2017**, *56* (34), 10070–10086.
