# Supplementary material for: Theoretical studies on anhydride dynamic covalent bond exchange mechanisms
Source: Commun Chem. 2025 Nov 17;8:351. doi: 10.1038/s42004-025-01736-3 (PMC12624005; doi:10.1038/s42004-025-01736-3)
Supplement: Supplementary file 3 — Description of Additional Supplementary Files [file 42004_2025_1736_MOESM3_ESM.pdf]

Description of Additional Supplementary Files

File name: Supplementary Data 1

Description: ZIP folder containing DFT-optimised .xyz files and IRC movie file.
